# Comparative Lung Bacterial Community Profiles in Pneumonic and Non-Pneumonic Akkaraman Lambs

**DOI:** 10.3390/ani16182871

**Published:** 2026-09-12

**Authors:** Zeynep Yerlikaya, Burak Karabulut, Eren Cankaya, Muhammed Girgin, Pelin Fatos Polat Dincer, Hatice Eroksuz

**Affiliations:** 1 Department of Microbiology, Faculty of Veterinary Medicine, Firat University, Elazig 23200, Türkiye; m.girgin@firat.edu.tr; 2 Department of Pathology, Faculty of Veterinary Medicine, Firat University, Elazig 23200, Türkiye; bkarabulut@firat.edu.tr (B.K.); ecankaya@firat.edu.tr (E.C.); heroksuz@firat.edu.tr (H.E.); 3 Department of Internal Medicine, Faculty of Veterinary Medicine, Dokuz Eylül University, Izmir 35400, Türkiye; pelinfatos.polat@deu.edu.tr

**Keywords:** 16S rRNA gene sequencing, bronchopneumonia, *Mannheimia*, microbial ecology, pathology, respiratory microbiota, small ruminant, veterinary microbiology

## Abstract

Ovine pneumonia is a multifactorial respiratory disease, yet bacterial community alterations associated with pathologically confirmed pneumonia in lambs remain poorly characterised. This study investigated the lung bacterial microbiota of non-pneumonic and pneumonic Akkaraman lambs by integrating gross and histopathological examination with 16S rRNA gene amplicon sequencing. The final dataset comprised lung samples from 15 non-pneumonic and 14 pneumonic lambs. Alpha diversity did not differ significantly between groups, whereas bacterial community composition differed significantly for both Bray–Curtis (*R*^2^ = 0.093, *p* = 0.001) and weighted UniFrac (*R*^2^ = 0.117, *p* = 0.006) distances, with no significant differences in multivariate dispersion. At the genus level, *Mannheimia* showed significantly higher relative abundance in pneumonic lungs after false-discovery-rate correction and was the only genus also identified as differentially abundant by ANCOM. *Mycoplasma*, *Fusobacterium*, and *Trueperella* showed greater relative representation in pneumonic lungs, whereas *Acinetobacter* and *Psychrobacter* showed the opposite pattern. Prevalence analysis further demonstrated contrasting taxon distributions between groups. Overall, naturally occurring pneumonia was associated with distinct alterations in lung bacterial community composition rather than a generalised loss of within-sample diversity, with *Mannheimia* representing the strongest pneumonia-associated genus-level signal.

## Simple Summary

Pneumonia is a complex respiratory disease in young lambs that develops through interactions among infectious agents, the host, and environmental factors. In this study, we examined lung tissues from non-pneumonic and pneumonic Akkaraman lambs to determine how lung bacterial communities differ during naturally occurring pneumonia. Pneumonia was confirmed by gross and histopathological examinations, and bacterial communities were characterised using 16S rRNA gene sequencing. The overall richness and diversity of bacterial taxa within individual lungs were similar between groups, but their composition and distribution differed between non-pneumonic and pneumonic lungs. *Mannheimia* showed the clearest association with pneumonia, while several other bacterial groups also differed between groups. These findings suggest that ovine pneumonia is associated with broader changes in the lung bacterial community, rather than a change affecting a single bacterial group. Combining pathological examination with bacterial community analysis may therefore provide a more complete understanding of pneumonia in lambs. It may support future studies aimed at improving the diagnosis and management of ovine respiratory disease.

## 1. Introduction

Pneumonia is a major health problem in young lambs and contributes substantially to morbidity, mortality, impaired growth, carcass losses, treatment costs, and reduced productivity in sheep production [1,2]. Beyond its economic consequences, respiratory disease represents an important animal health and welfare concern, particularly during early life, when severe pulmonary disease may compromise growth and survival. Clinical management can also be challenging because ovine pneumonia is a multifactorial disease arising from interactions among infectious agents, host susceptibility and immune status, environmental conditions, and management-related stressors [3,4]. Factors such as overcrowding, inadequate ventilation, transportation, adverse weather conditions, and concurrent viral or *Mycoplasma* infections may compromise respiratory defence mechanisms and increase susceptibility to pneumonia [5]. Consequently, effective diagnosis, treatment, and prevention require consideration of both pulmonary lesions and the broader host and management context in which pneumonia develops.

Bacterial pathogens remain important components of ovine respiratory disease, with members of the family Pasteurellaceae, particularly *Mannheimia haemolytica*, *Bibersteinia trehalosi*, and *Pasteurella multocida*, frequently implicated in pneumonic lambs and sometimes occurring as mixed infections [6]. However, culture-based isolation and targeted molecular assays may not fully characterise the broader bacterial community associated with pneumonic lung lesions. Pathology–bacteriology studies in lambs have demonstrated associations between pneumonic lesion patterns and bacterial agents, highlighting the importance of interpreting microbial findings alongside gross and histopathological evidence of disease [7]. Culture-independent 16S rRNA gene amplicon sequencing provides a complementary approach for characterising lung-associated bacterial communities beyond routinely cultured or specifically targeted organisms, although short-read 16S data have limited taxonomic resolution, particularly at the species level [8,9]. Moreover, because lung tissue represents a low-biomass microbial environment, sequencing-based profiles require careful consideration of contaminant DNA and workflow-related biases [10]. Integrating microbiome profiling with pathological characterisation may therefore provide a broader framework for investigating bacterial community alterations associated with pneumonia.

Despite growing interest in the respiratory microbiome, information on lung-associated bacterial communities in sheep, particularly in young lambs with naturally occurring pneumonia, remains limited. Previous studies have demonstrated inter-individual and anatomical-site variation in ovine respiratory bacterial communities and reported pneumonia-associated alterations involving taxa including *Mannheimia*, *Fusobacterium*, *Pasteurella*, *Mycoplasma*, and *Trueperella* [11,12,13,14]. However, the relationship between morphologically confirmed pneumonic lesions and the broader bacterial community structure of lamb lungs remains incompletely characterised, particularly with respect to community diversity, taxonomic composition, and the prevalence of bacterial taxa in pneumonic and non-pneumonic animals.

Therefore, the present study aimed to characterise and compare the lung bacterial microbiota of non-pneumonic and pneumonic Akkaraman lambs using 16S rRNA gene amplicon sequencing. Pneumonic cases were identified based on gross pathological lesions and confirmed by histopathological examination. Microbial diversity, taxonomic composition, genus-level differential abundance, and taxon prevalence patterns were evaluated to assess pneumonia-associated differences in the lung bacterial community.

## 2. Materials and Methods

### 2.1. Animals and Case Selection

Lung tissue samples were collected between January and June 2026 from 15–30-day-old Akkaraman lambs submitted for diagnostic necropsy to the Department of Pathology, Faculty of Veterinary Medicine, Firat University (Elazığ, Türkiye). During this period, a total of 106 sheep were submitted for diagnostic necropsy, of which 82 were younger than three months of age. Nineteen lambs showed gross pathological findings consistent with pneumonia; three were excluded because of marked autolysis or putrefaction, and one because it was of a different breed. Consequently, all 15 eligible pneumonic Akkaraman lambs were included in the study. For comparison, 15 non-pneumonic Akkaraman lambs within the same age range were selected from diagnostic necropsy cases that had died from confirmed extrapulmonary diseases and showed no gross or microscopic evidence of pneumonia. The same 15–30-day age range was applied to both groups to minimise potential confounding associated with age-related variation in lung bacterial community composition. Thus, the study comprised 30 lambs, with balanced pneumonic and non-pneumonic groups. Immediately after necropsy, lung tissue samples were collected using sterile instruments. Samples from pneumonic lungs were collected from representative areas of pulmonary consolidation, whereas non-pneumonic samples were collected from grossly normal lung parenchyma. Each sample was divided into two portions: one was fixed in 10% neutral-buffered formalin for pathological examination, and the other was stored in sterile tubes at −80 °C until DNA extraction for microbiome analysis.

### 2.2. Macroscopic and Histopathological Examination

All lungs were examined macroscopically at necropsy. Lobar pneumonia was diagnosed based on characteristic macroscopic findings, including pulmonary consolidation, hepatisation, enlargement, and increased firmness of the affected lobe, yellow mucopurulent exudate expressed from the small airways after gentle compression, and involvement of at least one pulmonary lobe. Representative lung tissue samples from all animals were routinely processed, embedded in paraffin, sectioned at 4 μm, and stained with haematoxylin and eosin (H&E) for histopathological examination. Pneumonia was histopathologically confirmed by the presence of inflammatory cells, inflammatory exudate, or both within the alveolar lumina. Samples assigned to the non-pneumonic group showed no histopathological evidence of pneumonia. Following diagnostic confirmation, pneumonic lesions were further classified according to a previously described modified classification system [7]. Lesions were categorised according to the duration of inflammation (acute, subacute, or chronic), the predominant histopathological pattern, and lesion severity.

### 2.3. DNA Extraction

Genomic DNA was extracted from approximately 25 mg of lung tissue by BM Labosis Laboratory (Ankara, Türkiye) using the GeneMATRIX Tissue & Bacterial DNA Purification Kit (EurX, Gdańsk, Poland) according to the manufacturer’s solid-tissue protocol. Tissue samples were cryogenically ground to a fine powder under liquid nitrogen using a pre-cooled mortar and pestle. The powdered tissue was transferred to a microcentrifuge tube, 350 µL of Lyse T buffer was added, and the suspension was thoroughly mixed. Samples were subsequently treated with 2 µL of RNase A and 20 µL of Proteinase K and incubated at 56 °C until complete tissue lysis. DNA was then purified using the kit’s spin-column procedure according to the manufacturer’s instructions. DNA concentration was quantified using a Qubit 3.0 Fluorometer and the Qubit dsDNA HS Assay Kit (Thermo Fisher Scientific, Waltham, MA, USA). The sequencing facility required a minimum pre–amplification genomic DNA concentration of ≥50 ng/µL for subsequent 16S rRNA gene amplicon library preparation.

### 2.4. 16S rRNA Gene Amplification and Sequencing

The V3–V4 region of the bacterial 16S rRNA gene was amplified using the locus-specific primers 341F (5′-CCTACGGGNGGCWGCAG-3′) and 785R (5′-GACTACHVGGGTATCTAATCC-3′) [15], generating an amplicon of approximately 460 bp. This primer pair is a previously validated V3–V4 bacterial primer set with broad bacterial taxonomic coverage and phylum representation and has been widely used for 16S rRNA gene-based microbial community profiling [15]. Amplicon library preparation and sequencing were performed by BM Labosis Laboratory (Ankara, Türkiye). Amplicon PCR was performed in a 25-µL reaction containing 2.5 µL of template DNA (5 ng/µL; 12.5 ng total), 5 µL each of the 1 µM forward and reverse primers, and 12.5 µL of 2× KAPA HotStart PCR Mix. Thermal cycling consisted of an initial denaturation at 95 °C for 3 min, followed by 25 cycles of 95 °C for 30 s, 55 °C for 30 s, and 72 °C for 30 s, with a final extension at 72 °C for 5 min. PCR products were purified using AMPure XP magnetic beads (Beckman Coulter, Brea, CA, USA).

Dual indexing was subsequently performed using the Nextera XT Index Kit (Illumina, San Diego, CA, USA) in a 50-µL reaction containing 5 µL of purified amplicon, 5 µL each of the N7xx and S5xx index primers, 25 µL of 2× KAPA HotStart PCR Mix (KAPA Biosystems, Wilmington, MA, USA), and 10 µL of nuclease-free water. Index PCR consisted of an initial denaturation at 95 °C for 3 min, followed by eight cycles of 95 °C for 30 s, 55 °C for 30 s, and 72 °C for 30 s, with a final extension at 72 °C for 5 min. Indexed libraries were purified using AMPure XP magnetic beads, quantified by real-time PCR, normalised, pooled, and sequenced on an Illumina NovaSeq 6000 platform (Illumina, San Diego, CA, USA) using 2 × 250-bp paired-end sequencing. A PCR negative control was included during library preparation to monitor potential contamination introduced during amplification, and no amplification band was observed in the negative control. Demultiplexed FASTQ files were provided by BM Labosis Laboratory and used for downstream bioinformatic analysis.

### 2.5. Bioinformatic Analysis

Demultiplexed paired-end FASTQ files were processed using QIIME 2 version 2022.11 [16]. Primer sequences were removed using Cutadapt v4.2 [17], and reads shorter than 200 bp after primer trimming were discarded using fastp v1.3.3 [18]. Amplicon sequence variants (ASVs) were inferred using the DADA2 paired-end denoising workflow [19]. Because primer sequences had already been removed using Cutadapt, no additional 5′ trimming was applied in DADA2. No fixed-length truncation was applied to either forward or reverse reads to retain the available read length and maximise the overlap required for merging the approximately 460-bp V3–V4 amplicon generated by 2 × 250-bp paired-end sequencing. The maximum expected error threshold was set to 4 for both forward and reverse reads, and the quality truncation threshold was set to 2. DADA2 performed quality filtering, denoising, paired-end merging, and chimera removal, and only successfully merged high-quality read pairs were retained for downstream analyses. Taxonomic assignment of representative ASV sequences was performed using the q2-feature-classifier plugin [20] with a Naïve Bayes classifier trained on the SILVA 138 (99%) reference database [21]. ASVs that remained unassigned or were classified only at the bacterial domain level were removed before downstream analyses. No dedicated bioinformatic contaminant-identification algorithm was applied. Multiple sequence alignment, masking, phylogenetic tree construction, and midpoint rooting were performed using the q2-phylogeny plugin with the align-to-tree-mafft-fasttree pipeline [22,23]. Diversity analyses were performed after rarefaction to 10,000 sequences per sample, a depth that standardised sequencing effort across samples while retaining all 29 samples in the dataset. Alpha diversity was assessed using observed ASVs, Shannon diversity, Faith’s phylogenetic diversity, and Pielou’s evenness. Beta diversity was evaluated using Bray–Curtis dissimilarity and weighted UniFrac distances and visualised using principal coordinates analysis (PCoA).

### 2.6. Statistical Analysis

Differences in each alpha-diversity metric between groups were assessed using the Kruskal–Wallis test, whereas beta diversity differences were evaluated separately for Bray–Curtis dissimilarity and weighted UniFrac distance matrices using PERMANOVA with 999 permutations. Homogeneity of multivariate dispersion between groups was assessed separately for both distance matrices using PERMDISP with 999 permutations to evaluate whether the observed between-group differences could be influenced by unequal within-group dispersion. Genus-level univariate relative-abundance comparisons were restricted to classified genera detected in at least 20% of samples and with an overall mean relative abundance of at least 0.01%. Retained genera were compared between groups using two-sided Mann–Whitney *U* tests with Benjamini–Hochberg false discovery rate correction [24]. These analyses were used to evaluate between-group differences in relative–abundance distributions and were interpreted in conjunction with ANCOM [25], which was also applied as a composition-aware differential abundance method. For ANCOM, the feature table was collapsed to the genus level and restricted to taxa detected in at least 20% of samples, after which features with fewer than 10 total counts across the dataset were removed. A pseudocount of 1 was then added before ANCOM analysis, which was performed within the QIIME 2 composition framework using pathological group as the grouping variable. A prevalence-based analysis was performed at the genus level, with taxa retained for visualisation if they were detected in at least 80% of samples in either study group and showing an absolute between-group prevalence difference of at least 20 percentage points. These thresholds were used for descriptive visualisation and did not represent criteria for statistical significance. Additional statistical analyses and publication-quality figures were generated in a Python 3.9.12 environment using pandas (v1.5.3), NumPy (v1.23.5), SciPy (v1.10.1), Matplotlib (v3.7.2), and statsmodels (v0.14.0). Statistical significance was set at *p* < 0.05, and false-discovery-rate-adjusted *p*-values were reported as *q*-values where applicable.

## 3. Results

### 3.1. Pneumonic Lungs Exhibited a Spectrum of Gross Lesions, with Predominantly Acute Bronchopneumonia

**Figure 1:**
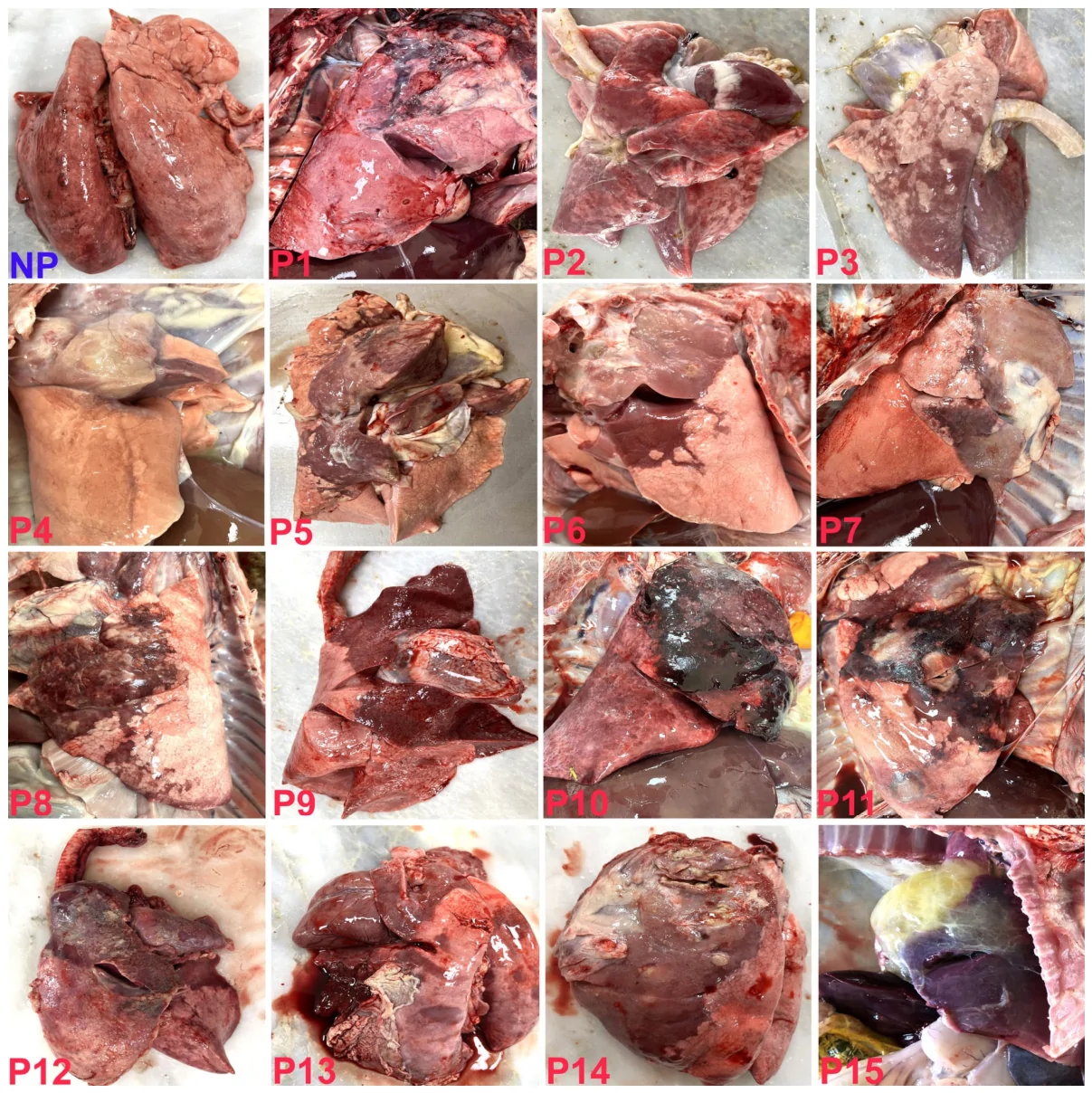
Gross appearance of non-pneumonic and pneumonic lamb lungs. NP, non-pneumonic lung; P1–P15, representative pneumonic lungs. Pneumonic lungs were qualitatively grouped according to the extent and severity of gross lesions as mild (P1–P3), moderate (P4–P7), severe (P8–P11), and very severe (P12–P15).

Gross pathological examination revealed variable lesion severity among pneumonic lungs. For descriptive presentation, pneumonic cases were qualitatively grouped according to the extent and severity of gross lesions as mild (P1–P3), moderate (P4–P7), severe (P8–P11), or very severe (P12–P15) (Figure 1). The cranial lung lobes were the most frequently affected and were characterised by varying degrees of consolidation accompanied by dark-red to dark-purple discolouration. In the most severely affected lungs, the pleural surface was covered by fibrinous or fibrinopurulent exudate, with multifocal abscesses and focal areas of necrosis also observed (Figure 1).

**Figure 2:**
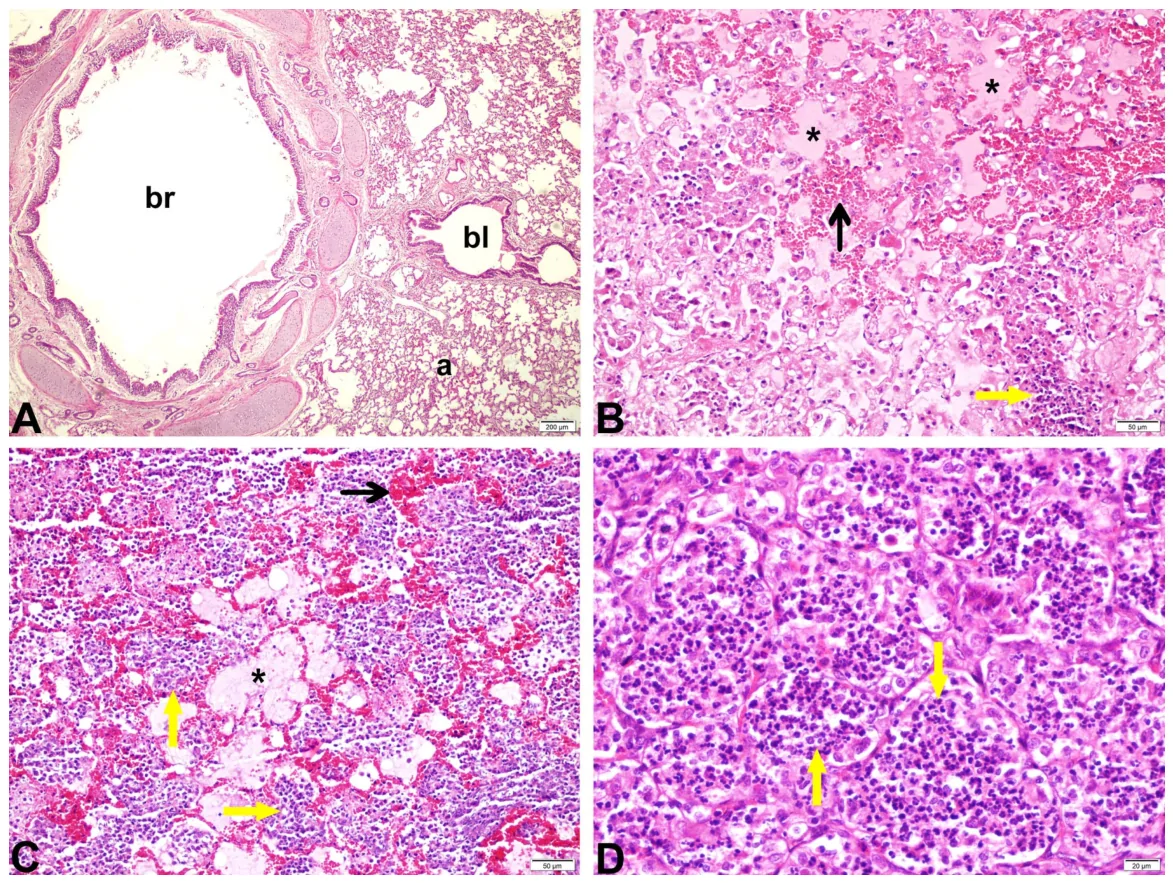
Histopathological features of non-pneumonic and pneumonic lamb lungs. (**A**) Non-pneumonic lung showing normal pulmonary architecture with bronchi (br), bronchioles (bl), and alveoli (a). (**B**) Acute catarrhal bronchopneumonia characterised by hyperaemic alveolar capillaries (black arrow), mild neutrophilic infiltration (yellow arrow), and intra-alveolar oedema (asterisks). (**C**) Catarrhal purulent bronchopneumonia showing hyperaemic alveolar capillaries (black arrow), neutrophilic infiltration within alveolar lumina (yellow arrows), and intra-alveolar oedema (asterisk). (**D**) Purulent bronchopneumonia characterised by extensive neutrophilic infiltration within alveolar lumina (yellow arrows). Haematoxylin and eosin (H&E) stain. Original magnification: (**A**) ×40; (**B**,**C**) ×200; (**D**) ×400.

**Figure 3:**
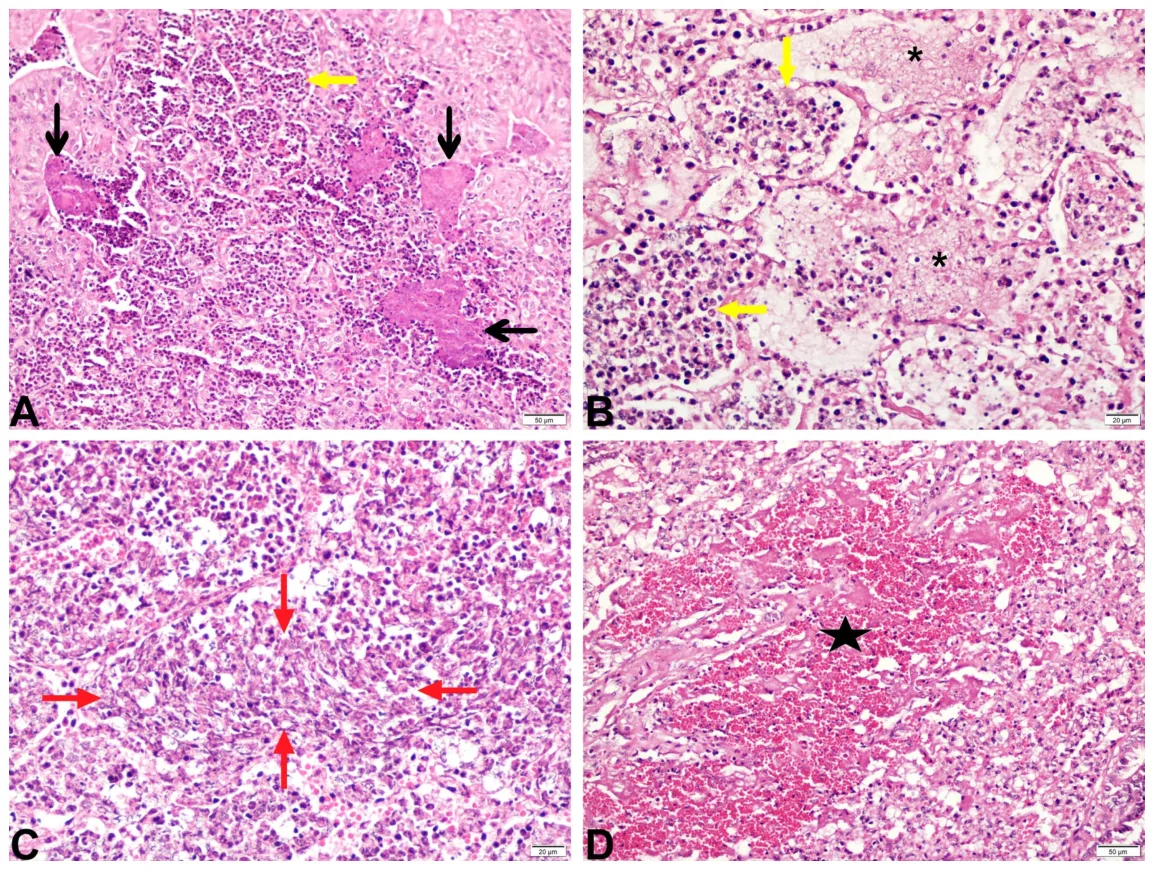
Histopathological features of advanced pneumonic lesions in lamb lungs. (**A**) Purulent necrotic bronchopneumonia characterised by eosinophilic necrotic debris (black arrows) and neutrophilic infiltration within alveolar lumina (yellow arrow). (**B**) Fibrinopurulent bronchopneumonia showing neutrophilic infiltration within alveolar lumina (yellow arrows) and intra-alveolar fibrin deposition (asterisks). (**C**) Fibrinous pneumonia characterised by clusters of oat cells within the alveolar lumina (region between the red arrows). (**D**) Fibrinohaemorrhagic bronchopneumonia showing extensive haemorrhage (star). Haematoxylin and eosin (H&E) stain. Original magnification: (**A**,**D**) ×200; (**B**,**C**) ×400.

Histopathological examination confirmed pneumonia in all pneumonic cases, with acute inflammatory lesions predominating. Histopathological patterns included acute catarrhal bronchopneumonia (*n* = 3; Figure 2B), catarrhal purulent bronchopneumonia (*n* = 3; Figure 2C), purulent bronchopneumonia (*n* = 1; Figure 2D), purulent necrotic bronchopneumonia (*n* = 2; Figure 3A), fibrinohaemorrhagic bronchopneumonia (*n* = 2; Figure 3D), fibrinopurulent bronchopneumonia (*n* = 3; Figure 3B), and fibrinous pneumonia characterised by oat-cell formation (*n* = 1; Figure 3C).

The principal histopathological findings included alveolar capillary hyperaemia, intra-alveolar oedema, neutrophilic infiltration, fibrin deposition, haemorrhage, multifocal necrosis, and oat-cell formation. In contrast, non-pneumonic lungs showed no gross or microscopic evidence of inflammatory pulmonary lesions (Figure 1, Figure 2 and Figure 3).

### 3.2. Pneumonia Was Associated with Altered Community Composition but Not Alpha Diversity

Library preparation was unsuccessful for one pneumonic sample (P14), resulting in 29 samples (15 non-pneumonic and 14 pneumonic) available for sequencing and downstream microbiome analyses. A total of 2,708,855 paired-end reads entered the DADA2 workflow, of which 1,776,928 non-chimeric reads were retained after denoising, paired-end merging, and chimera removal. DADA2 generated 30,646 ASVs prior to taxonomic filtering. Following the removal of unassigned features and features classified only at the bacterial domain level, the final dataset contained 4433 ASVs and 1,299,088 reads. All 29 samples were retained at a rarefaction depth of 10,000 reads per sample. Detailed sequencing and bioinformatic processing statistics are summarised in Supplementary Table S1.

**Figure 4:**
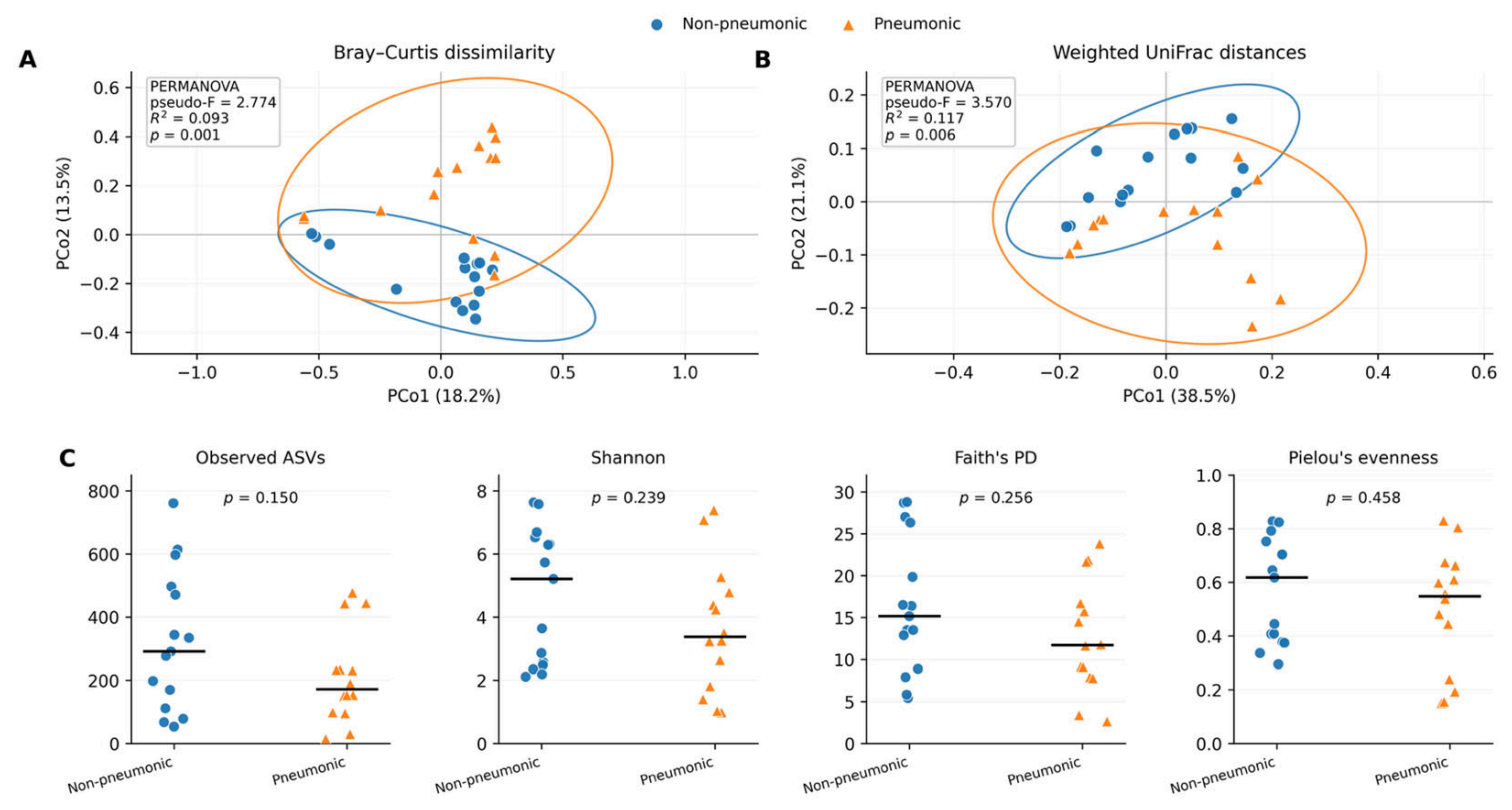
Alpha and beta diversity of lung bacterial communities in non-pneumonic and pneumonic lambs. (**A**) Principal coordinates analysis (PCoA) based on Bray–Curtis dissimilarity and (**B**) weighted UniFrac distances. Circles and triangles represent non-pneumonic and pneumonic samples, respectively. Ellipses represent 95% concentration ellipses based on the distribution of samples within each group. PERMANOVA statistics based on 999 permutations are shown within each panel. (**C**) Alpha-diversity indices, including observed ASVs, Shannon diversity, Faith’s phylogenetic diversity (PD), and Pielou’s evenness. Individual observations are shown, with horizontal black bars indicating group medians. Alpha-diversity comparisons were performed using Kruskal–Wallis tests.

Alpha-diversity analyses revealed no significant differences between non-pneumonic and pneumonic lungs in observed ASVs (*p* = 0.150), Shannon diversity (*p* = 0.239), Faith’s phylogenetic diversity (*p* = 0.256), or Pielou’s evenness (*p* = 0.458) (Figure 4C).

In contrast, beta-diversity analyses demonstrated significant differences in microbial community composition between non-pneumonic and pneumonic lungs. Principal coordinates analysis (PCoA) based on both Bray–Curtis dissimilarity and weighted UniFrac distances demonstrated partial separation between the two groups (Figure 4A,B). PERMANOVA confirmed significant differences in community composition based on both Bray–Curtis dissimilarity (pseudo-*F* = 2.774, *R*^2^ = 0.093, *p* = 0.001) and weighted UniFrac distances (pseudo-*F* = 3.570, *R*^2^ = 0.117, *p* = 0.006). PERMDISP revealed no significant differences in multivariate dispersion between groups for either Bray–Curtis (*p* = 0.883) or weighted UniFrac (*p* = 0.142), indicating that the significant PERMANOVA results were not attributable to differences in within-group dispersion.

### 3.3. Taxonomic Composition Differed Between Non-Pneumonic and Pneumonic Lungs, with Mannheimia Emerging as the Principal Differentially Abundant Genus

**Figure 5:**
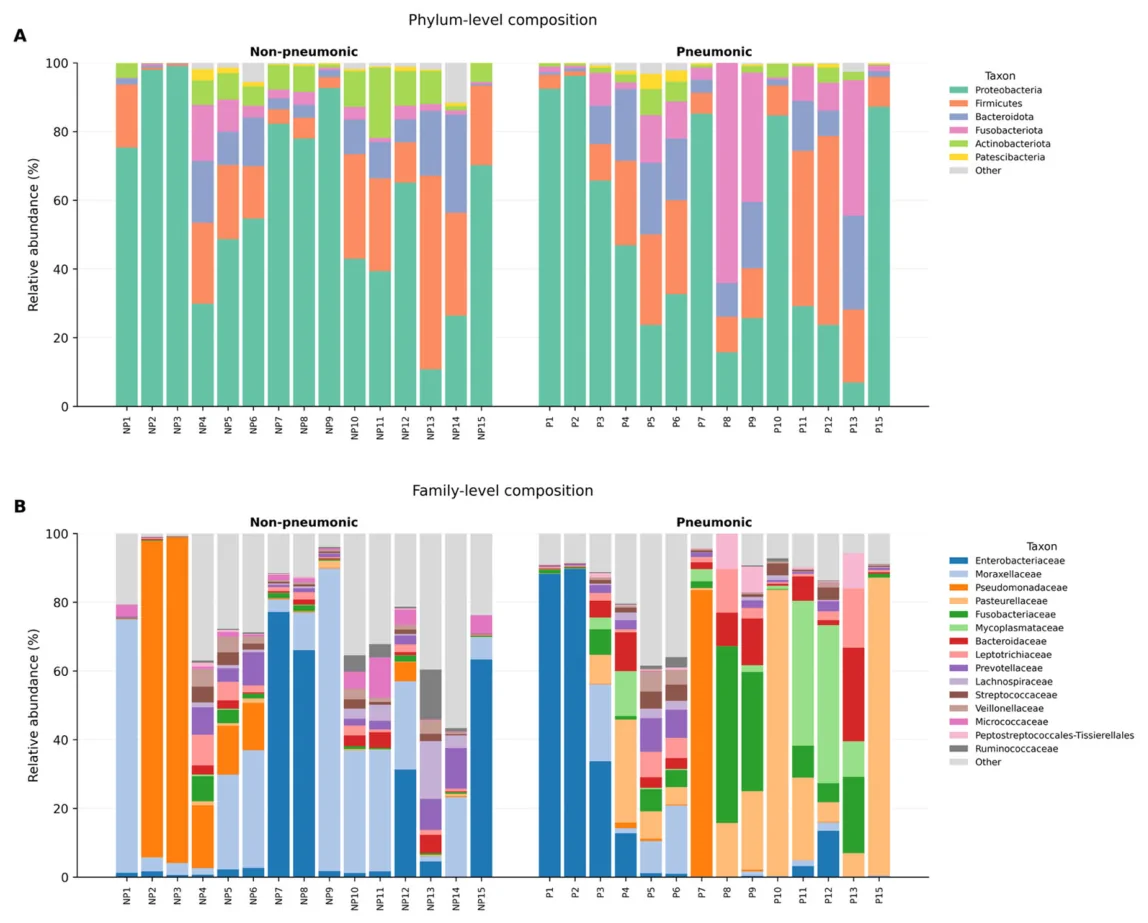
Descriptive taxonomic composition of bacterial communities in non-pneumonic and pneumonic lamb lungs. (**A**) Phylum-level and (**B**) family-level relative abundance profiles. Samples are grouped according to pathological status, with the groups separated by a visual gap. Non-pneumonic samples are labelled NP1–NP15, whereas pneumonic samples are labelled according to the corresponding gross pathological specimens shown in Figure 1. One pneumonic sample (P14) was excluded because library preparation was unsuccessful and was therefore not included in downstream microbiome analyses. Low-abundance taxa are grouped as Other.

Taxonomic profiling revealed differences in bacterial community composition between non-pneumonic and pneumonic lungs (Figure 5). At the phylum level, Proteobacteria was the dominant lineage in both groups, with a higher mean relative abundance in non-pneumonic than in pneumonic lungs (60.9% vs. 51.1%). Firmicutes showed comparable mean proportions between groups (18.1% vs. 18.9%), whereas Fusobacteriota represented a greater proportion of the pneumonic lung microbiota (14.6% vs. 3.3%). Bacteroidota also showed a higher mean relative abundance in pneumonic lungs (11.3% vs. 8.6%), whereas Actinobacteriota was more highly represented in non-pneumonic lungs (6.5% vs. 2.3%) (Figure 5A). At the family level, non-pneumonic lungs were predominantly represented by Moraxellaceae (25.1%), Enterobacteriaceae (17.1%), and Pseudomonadaceae (16.0%), whereas pneumonic lungs showed greater mean relative abundances of Pasteurellaceae (21.3%), Enterobacteriaceae (17.4%), Fusobacteriaceae (10.6%), Mycoplasmataceae (8.8%), and Bacteroidaceae (6.1%) (Figure 5B).

**Figure 6:**
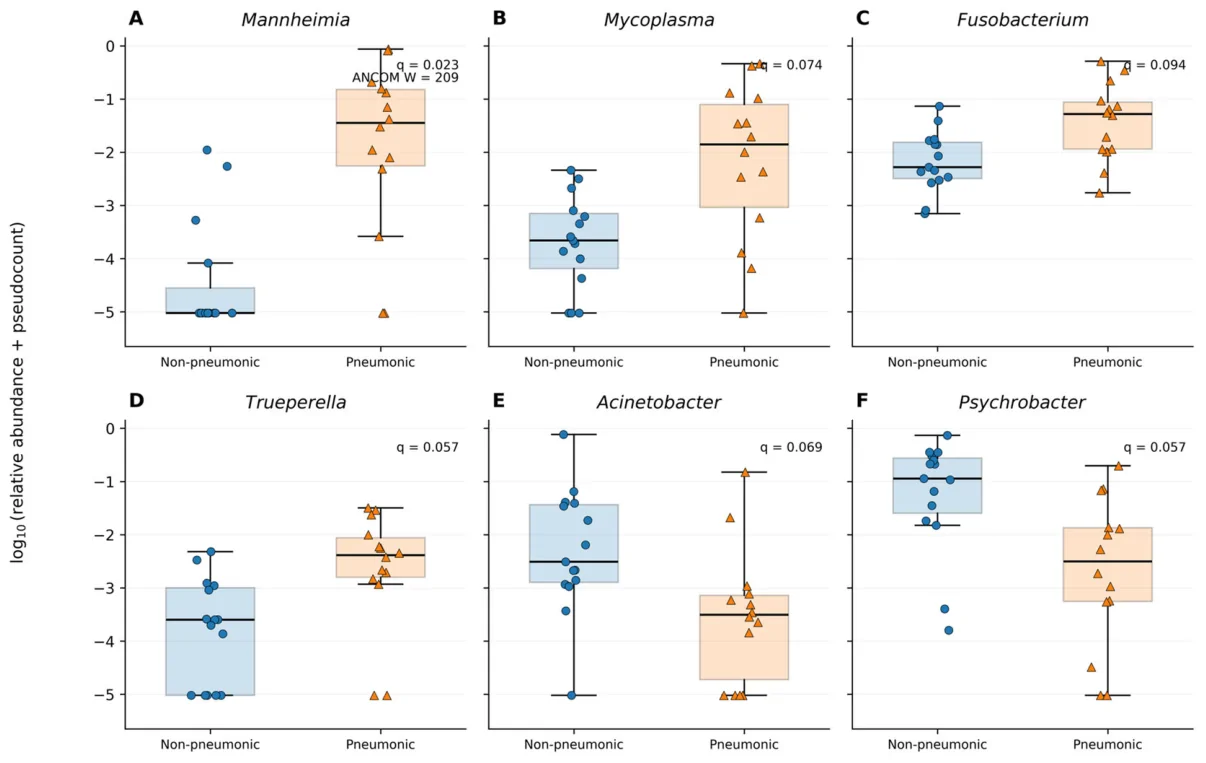
Genus-level relative abundance profiles of selected bacterial taxa in non-pneumonic and pneumonic lamb lungs. (**A**) *Mannheimia*; (**B**) *Mycoplasma*; (**C**) *Fusobacterium*; (**D**) *Trueperella*; (**E**) *Acinetobacter*; (**F**) *Psychrobacter*. Relative abundances were visualised after log10(relative abundance + pseudocount) transformation for display purposes only. Statistical comparisons were performed using two-sided Mann–Whitney *U* tests with Benjamini–Hochberg false-discovery-rate correction. False-discovery-rate-adjusted *p*-values (*q*-values) are shown within each panel. *Mannheimia* was additionally identified as differentially abundant by ANCOM (*W* = 209). The taxa shown were selected for visualisation to illustrate prominent between-group abundance patterns; selection for display did not influence statistical testing or false-discovery-rate correction. Complete genus-level results are provided in Supplementary Table S2, with ANCOM results in Supplementary Table S3.

Genus-level comparisons further characterised these compositional differences (Figure 6, Table S2). *Glutamicibacter* showed the strongest FDR-corrected univariate difference (*q* = 0.007), and was detected predominantly in non-pneumonic samples, whereas *Mannheimia* showed a significantly higher relative abundance in pneumonic lungs (*q* = 0.023). Of these taxa, only *Mannheimia* was additionally identified as differentially abundant by ANCOM (*W* = 209; Table S3). Several other genera showed directional differences in relative abundance, including higher relative abundances of *Mycoplasma*, *Fusobacterium*, and *Trueperella* in pneumonic lungs and lower relative abundances of *Acinetobacter* and *Psychrobacter*; however, these comparisons did not remain significant at the 5% FDR threshold.

### 3.4. Pneumonia Was Associated with Shifts in the Prevalence of High-Prevalence Lung Taxa

**Figure 7:**
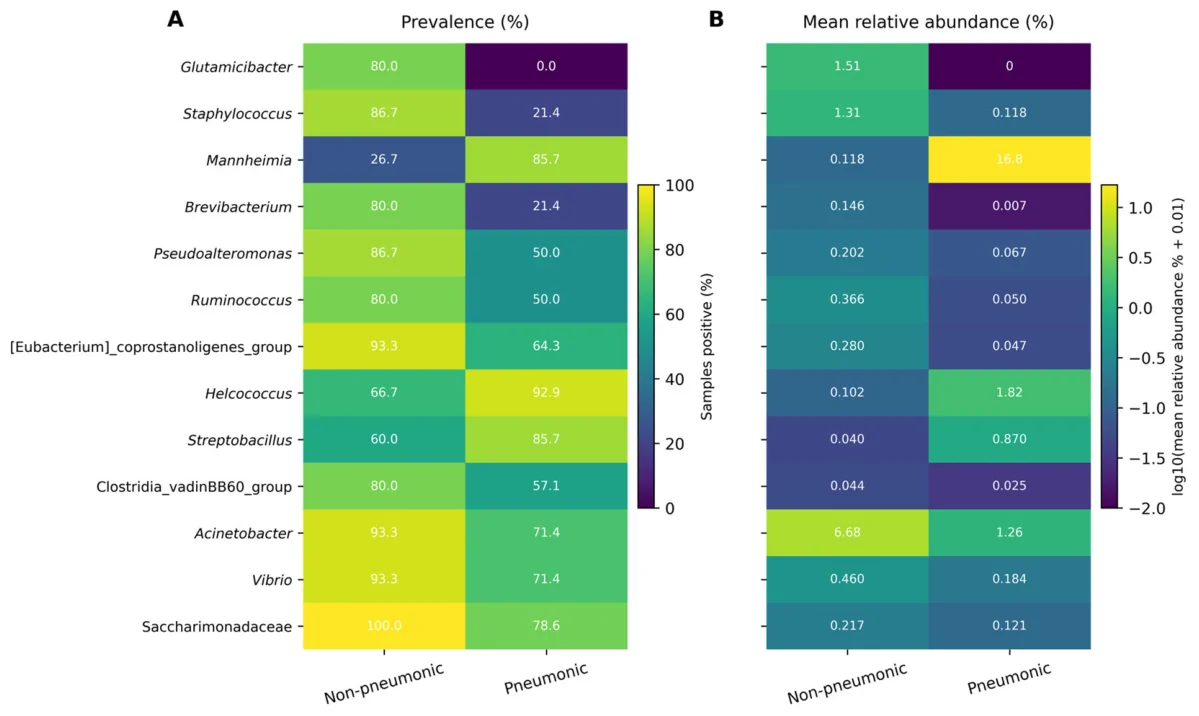
Prevalence and mean relative abundance patterns of high-prevalence bacterial taxa in non-pneumonic and pneumonic lamb lungs. Taxa were retained for visualisation when they were detected in at least 80% of samples in either study group and showed an absolute between-group prevalence difference of at least 20 percentage points (non-pneumonic, *n* = 15; pneumonic, *n* = 14). (**A**) Prevalence of the selected taxa in non-pneumonic and pneumonic lungs. Cell values indicate the percentage of samples in which each taxon was detected. (**B**) Group-wise mean relative abundance of the same taxa. Cell values indicate the untransformed mean relative abundance (%), whereas the colour scale represents the log10-transformed mean relative abundance following the addition of a pseudocount of 0.01 for visualisation.

Prevalence-based analysis revealed marked differences in high-prevalence bacterial taxa between non-pneumonic and pneumonic lungs (Figure 7). Thirteen taxa met the visualisation criteria of ≥80% prevalence in at least one study group and an absolute between-group prevalence difference of ≥20 percentage points. Among these, *Mannheimia* showed a prominent pneumonia-associated difference, being detected in 85.7% of pneumonic lungs compared with 26.7% of non-pneumonic lungs. This difference in prevalence was accompanied by a higher mean relative abundance in pneumonic lungs (16.8% vs. 0.1%).

Conversely, several high-prevalence taxa showed lower prevalence in pneumonic lungs. *Glutamicibacter* was detected in 80.0% of non-pneumonic samples but was not detected in pneumonic samples, whereas the prevalence of *Staphylococcus* decreased from 86.7% in non-pneumonic lungs to 21.4% in pneumonic lungs. Additional taxa also differed in prevalence between groups, indicating broader differences in the distribution of high-prevalence members of the lung bacterial community (Figure 7). Overall, these findings indicate that pneumonia was associated with shifts in the prevalence patterns of selected high-prevalence lung taxa.

## 4. Discussion

The present study demonstrated that naturally occurring lobar pneumonia in young Akkaraman lambs was associated with significant alterations in lung bacterial community composition compared with those in morphologically non-pneumonic lungs. Although alpha diversity did not differ significantly between groups, beta-diversity analyses demonstrated significant differences in community composition. Taxonomic profiling and prevalence-based analyses further supported this community-level restructuring, with *Mannheimia* emerging as the most consistently supported pneumonia-associated genus, as identified by both false-discovery-rate-corrected genus-level comparisons and ANCOM.

A notable finding was that pneumonia-associated differences in community composition occurred without significant changes in alpha diversity. This pattern reflects differences in the identity and relative representation of bacterial taxa rather than a generalised loss of within-sample richness, evenness, or phylogenetic diversity. Alterations in respiratory microbial community composition have also been reported in pneumonia and other respiratory diseases [13,26,27,28]. However, alpha-diversity findings across respiratory diseases remain heterogeneous, with the direction and magnitude of within-sample diversity differences varying among diseases and studies [29,30]. Importantly, the absence of significant differences in multivariate dispersion indicated that the PERMANOVA results were unlikely to be attributable to unequal within-group dispersion [31]. Together, these findings support an ecological interpretation in which pneumonia is associated with reorganisation of the lung bacterial community rather than a uniform decline in microbial diversity. 

Interpretation of lung microbiome data is strengthened when bacterial community profiles are considered alongside gross and histopathological findings rather than bacterial detection alone [32]. Although culture-independent 16S rRNA gene sequencing provides a broad overview of lung-associated bacterial communities, it cannot distinguish viable from non-viable microorganisms and has limited taxonomic resolution, particularly at the species level. Moreover, sequence detection alone cannot establish whether a detected taxon is transiently present, persists within the respiratory tract, or actively contributes to pulmonary disease [33,34]. Integrating microbiome profiling with pathological examination therefore provides a biological framework for interpreting community alterations in morphologically verified non-pneumonic and pneumonic lung tissues. This approach is consistent with ecological models of the lung microbiome, which emphasise that sequence-based profiles should be interpreted within the biological and disease context of the respiratory tract rather than as independent evidence of causality [33,35,36]. Accordingly, the present findings demonstrate pneumonia-associated microbial community alterations but do not establish that individual bacterial taxa caused the observed pulmonary lesions.

Taxonomic profiling further supported broader restructuring of the lung bacterial community in association with pneumonia. Proteobacteria remained the dominant phylum in both groups, whereas Fusobacteriota and Bacteroidota showed greater mean relative representation in pneumonic lungs. At the family level, Pasteurellaceae, Fusobacteriaceae, Mycoplasmataceae, and Bacteroidaceae showed greater mean relative representation in pneumonic lungs, while Moraxellaceae and Pseudomonadaceae were more prominent in non-pneumonic lungs. Similar pneumonia-associated compositional patterns have been reported in sheep. Miao et al. described marked alterations in the lung microbiota of pneumonic Hu sheep, including prominent representation of Pasteurellaceae, Fusobacteriaceae, Bacteroidaceae, and Mycoplasmataceae and disease severity-associated shifts involving *Mannheimia* and *Fusobacterium* [13]. Similarly, Feng et al. reported restructuring of the pulmonary microbiota in mycoplasmal pneumonia involving *Mycoplasma*, *Mannheimia*, *Pasteurella*, *Fusobacterium*, *Trueperella*, and other bacterial taxa [14]. More broadly, disease-associated alterations in respiratory bacterial community composition have also been reported in other animal species, including cattle and horses, although respiratory microbiota are influenced by host, anatomical, environmental, and methodological factors [37,38]. Taken together, these observations support the interpretation that ovine pneumonia is associated with broader compositional restructuring rather than a uniform taxonomic shift dominated by a single bacterial lineage. However, because the phylum- and family-level profiles in the present study were descriptive, visually apparent differences in relative abundance should not be interpreted as statistically confirmed enrichment or depletion.

Within this broader compositional shift, *Mannheimia* emerged as the most consistently supported pneumonia-associated genus, showing higher relative abundance in pneumonic lungs in the FDR-corrected analysis and being the only genus additionally identified by ANCOM. *Mycoplasma*, *Fusobacterium*, and *Trueperella* also showed substantial increases in relative representation in pneumonic lungs. These patterns are biologically plausible and are consistent with previous ovine respiratory studies in which *Mannheimia*, *Mycoplasma*, *Fusobacterium*, and *Trueperella* have been prominent components of pneumonic lung communities, often in the context of polymicrobial infection [13,14,32,39,40].

In particular, *Fusobacterium* has been reported at high relative abundance in severe ovine pneumonia, whereas *Mycoplasma* and *Trueperella* have also been enriched in diseased sheep lungs [13,14]. Although differences in these additional genera were not statistically significant after FDR correction and were not identified by ANCOM, this does not necessarily indicate the absence of a biologically relevant signal. Rather, the concordance of their abundance patterns with broader taxonomic patterns and previous respiratory studies suggests that they may form part of the broader pneumonia-associated community restructuring. Conversely, *Acinetobacter* and *Psychrobacter* showed greater relative representation in non-pneumonic lungs. Both genera have been reported as common members of respiratory microbial communities in other animal species, including apparently healthy cattle [41,42], but their ecological significance in non-pneumonic ovine lung remains uncertain. Their contrasting distribution should therefore be interpreted as part of the overall community shift rather than as evidence of a protective or health-associated role.

Prevalence analysis provided a complementary perspective by showing that differences between non-pneumonic and pneumonic lungs involved not only relative abundance but also the frequency with which individual taxa were detected across animals. *Mannheimia* was detected in a greater proportion of pneumonic lungs, whereas *Glutamicibacter* and *Staphylococcus*, both frequently detected in non-pneumonic lungs, were markedly less prevalent. The particularly contrasting distribution of *Glutamicibacter* is noteworthy given its significant FDR-corrected univariate difference, although its ecological significance in the ovine lung remains unknown. More broadly, these prevalence patterns suggest that pneumonia was associated with differences in the distribution of multiple bacterial populations across animals, complementing the abundance-based findings. High prevalence in non-pneumonic lungs, however, should not be interpreted as evidence of a stable or protective “healthy core”, particularly given the substantial inter-individual and spatial variability previously reported in the sheep lung microbiota [11].

Vaccination and farm management may represent additional factors influencing pneumonia-associated bacterial patterns in lambs. Vaccination programmes against ovine respiratory complex target major bacterial pathogens, including *Mannheimia haemolytica*, *Bibersteinia trehalosi*, and *Pasteurella multocida*, and may begin during the first weeks of life with subsequent booster immunisation [43,44]. Given the young age of the lambs examined here (15–30 days), differences in vaccination status, stage of primary immunisation, or maternally derived immunity could have influenced susceptibility to respiratory infection; however, these data were not available. Farm-management factors such as housing, stocking density, ventilation, climatic exposure, transportation, and other stressors may likewise affect respiratory disease susceptibility and bacterial transmission [1,2,43,44], potentially contributing to between-farm variation in the prevalence and relative representation of respiratory taxa. As detailed farm-level management data were not available, these relationships remain hypothetical and should be examined in future studies integrating vaccination, husbandry, and microbiome data.

An important strength of this study is the integration of gross and histopathological confirmation with community-level microbiome profiling, supported by complementary analyses of microbial diversity, taxonomic composition, differential abundance, and prevalence. This pathology-informed design provides a framework for interpreting bacterial community differences within morphologically defined lung disease. Future studies incorporating larger cohorts, longitudinal sampling, and higher-resolution taxonomic and functional approaches will be important for determining the reproducibility of these patterns across populations and for resolving species-level associations and their relationships with lesion severity.

Several limitations should be considered when interpreting these findings. The relatively small sample size may have limited statistical power to detect moderate taxon-level differences, whereas the cross-sectional post-mortem design precludes assessment of temporal relationships between microbial community changes and pneumonia development. In addition, because the non-pneumonic lambs died from extrapulmonary diseases despite having no gross or histopathological evidence of pneumonia, they should be considered morphologically non-pneumonic reference cases rather than systemically healthy controls. Although these animals showed no evidence of pulmonary inflammation, an indirect influence of their underlying extrapulmonary diseases on lung bacterial community composition cannot be excluded. The use of V3–V4 16S rRNA gene amplicon sequencing also entails inherent limitations, including primer-dependent amplification bias and limited taxonomic resolution, particularly at the species and strain levels. Compared with shotgun metagenomic sequencing, this approach does not provide direct information on microbial gene content or functional potential and may therefore overlook functionally relevant differences within bacterial communities.

## 5. Conclusions

Naturally occurring lobar pneumonia in young Akkaraman lambs was associated with distinct restructuring of the lung bacterial community rather than a generalised loss of within-sample diversity. Although alpha diversity did not differ significantly between groups, beta-diversity analyses demonstrated significant differences in community composition, without evidence that these differences were driven by unequal multivariate dispersion. *Mannheimia* represented the most consistently supported pneumonia-associated genus, while abundance and prevalence analyses indicated broader shifts involving multiple bacterial taxa. Integrating 16S rRNA gene profiling with gross and histopathological confirmation strengthened the biological interpretation of these community-level differences within morphologically confirmed pulmonary disease. These findings describe associations and do not establish a causal or pathogenic role for individual bacterial taxa. Larger, longitudinal studies incorporating higher-resolution taxonomic and functional approaches, together with relevant vaccination and farm-management information, will be important for determining the reproducibility and clinical significance of these pneumonia-associated microbial patterns.

## Data Availability

The raw 16S rRNA gene amplicon sequencing data generated in this study have been deposited in the NCBI Sequence Read Archive (SRA) under BioProject accession number PRJNA1510795. Additional data supporting the findings of this study are available within the article and its Supplementary Materials. Further information or additional data may be obtained from the corresponding author upon reasonable request.

## Supplementary Materials

The following supporting information can be downloaded at https://www.mdpi.com/article/10.3390/ani16182871/s1, Table S1: Summary of sequencing output, DADA2 processing, and final dataset characteristics used for downstream analyses. Table S2: Complete genus-level differential abundance analysis between non-pneumonic and pneumonic lamb lungs; Table S3: Complete ANCOM differential abundance results.

## Abbreviations

The following abbreviations are used in this manuscript:

ANCOM	Analysis of Composition of Microbiomes
ASV	Amplicon Sequence Variant
DADA2	Divisive Amplicon Denoising Algorithm 2
FDR	False discovery rate
NCBI	National Center for Biotechnology Information
PCoA	Principal coordinates analysis
PD	Phylogenetic diversity
PERMANOVA	Permutational multivariate analysis of variance
PERMDISP	Permutational analysis of multivariate dispersions
QIIME 2	Quantitative Insights Into Microbial Ecology 2
SRA	Sequence Read Archive
