# Supplementary material for: Comparative Lung Bacterial Community Profiles in Pneumonic and Non-Pneumonic Akkaraman Lambs
Source: Animals (Basel). 2026 Sep 12;16(18):2871. doi: 10.3390/ani16182871 (PMC13603732; doi:10.3390/ani16182871)
Supplement: Supplementary file 1 [file animals-16-02871-s001.zip › table s1.pdf]

Table S1. Summary of sequencing output, DADA2 processing, and final dataset characteristics used for downstream analyses.

Analysis Step	Parameter	Value
**Sequencing input**	Samples included in downstream analyses	29
	Read pairs entering DADA2, total	2,708,855
	Read pairs per sample, median (range)	89,763 (50,695-172,346)
**DADA2 processing**	Quality-filtered reads	2,579,792 (95.2%)
	Denoised reads	2,409,864 (89.0%)
	Merged reads	2,115,509 (78.1%)
	Non-chimeric reads	1,776,928 (65.6%)
**Feature table**	ASVs generated by DADA2	30,646
**Final dataset used for downstream analyses**	ASVs retained after taxonomic filtering	4,433
	Reads retained in the final feature table	1,299,088
	Rarefaction depth	10,000 reads/sample
	Samples retained after rarefaction	29/29 (100%)
