# Supplementary material for: Comparative Lung Bacterial Community Profiles in Pneumonic and Non-Pneumonic Akkaraman Lambs
Source: Animals (Basel). 2026 Sep 12;16(18):2871. doi: 10.3390/ani16182871 (PMC13603732; doi:10.3390/ani16182871)
Supplement: Supplementary file 1 [file animals-16-02871-s001.zip › Supplementary_Materials_Tables_S2_S3_.pdf]

Supplementary Materials

Table S2. Complete genus-level differential abundance analysis between non-pneumonic and pneumonic lamb lungs.

Genera were retained when detected in at least 20% of samples and when overall mean relative abundance was ≥0.01%. Two-sided Mann–Whitney U tests were adjusted using the Benjamini–Hochberg procedure. Positive rank-biserial values indicate higher abundance in pneumonic lungs. Non-pneumonic, n = 15; pneumonic, n = 14.

Genus	Non-pneumonic mean (%)	Pneumonic mean (%)	Non-pneumonic median (%)	Pneumonic median (%)	Non-pneumonic prevalence (%)	Pneumonic prevalence (%)	Log2 fold change	Mann–Whitney U	p value	BH-adjusted q value	Rank-biserial effect size	Interpretation
Glutamicibacter	1.4910	0.0000	0.2487	0.0000	80.0	0.0	-10.612	21	4.564e-05	0.0074	-0.800	Significant (q < 0.05)
Mannheimia	0.1137	16.7508	0.0000	3.5942	26.7	85.7	7.190	185	2.764e-04	0.0225	0.762	Significant (q < 0.05)
Staphylococcus	1.2961	0.1171	0.1482	0.0000	86.7	21.4	-3.458	33	0.0011	0.0569	-0.686	Exploratory trend (0.05 ≤ q < 0.10)
Trueperella	0.0826	0.8668	0.0242	0.4130	66.7	85.7	3.377	174	0.0026	0.0569	0.657	Exploratory trend (0.05 ≤ q < 0.10)
Psychrobacter	18.5031	2.7514	11.3283	0.3573	100.0	85.7	-2.749	36	0.0028	0.0569	-0.657	Exploratory trend (0.05 ≤ q < 0.10)
Brevibacterium	0.1440	0.0067	0.0383	0.0000	80.0	21.4	-4.250	36.5	0.0016	0.0569	-0.652	Exploratory trend (0.05 ≤ q < 0.10)
Jeotgalicoccus	0.1550	0.0012	0.0717	0.0000	60.0	7.1	-6.171	45	0.0022	0.0569	-0.571	Exploratory trend (0.05 ≤ q < 0.10)
Brachybacterium	0.1283	0.0000	0.0687	0.0000	53.3	0.0	-7.083	49	0.0021	0.0569	-0.533	Exploratory trend (0.05 ≤ q < 0.10)
Acinetobacter	6.5519	1.2490	0.3085	0.0305	93.3	71.4	-2.390	39	0.0042	0.0693	-0.629	Exploratory trend (0.05 ≤ q < 0.10)
Streptobacillus	0.0390	0.8691	0.0117	0.1722	60.0	85.7	4.444	169	0.0051	0.0693	0.610	Exploratory trend (0.05 ≤ q < 0.10)
Planococcus	0.0250	0.0000	0.0000	0.0000	46.7	0.0	-4.764	56	0.0048	0.0693	-0.467	Exploratory trend (0.05 ≤ q < 0.10)
Tessaracoccus	0.0229	0.0000	0.0000	0.0000	46.7	0.0	-4.646	56	0.0048	0.0693	-0.467	Exploratory trend (0.05 ≤ q < 0.10)
Mycoplasma	0.0839	8.7458	0.0210	1.4825	80.0	92.9	6.687	168.5	0.0059	0.0741	0.605	Exploratory trend (0.05 ≤ q < 0.10)
Peptoniphilus	0.0141	0.0920	0.0000	0.0306	40.0	78.6	2.631	164.5	0.0076	0.0881	0.567	Exploratory trend (0.05 ≤ q < 0.10)
Peptostreptococcus	0.0208	0.2430	0.0000	0.0647	40.0	78.6	3.486	163.5	0.0087	0.0940	0.557	Exploratory trend (0.05 ≤ q < 0.10)
Fusobacterium	1.3864	10.5557	0.5220	5.2375	100.0	100.0	2.928	164	0.0107	0.0945	0.562	Exploratory trend (0.05 ≤ q < 0.10)
Helcococcus	0.1007	1.8191	0.0333	0.2084	66.7	92.9	4.162	163.5	0.0110	0.0945	0.557	Exploratory trend (0.05 ≤ q < 0.10)
Nosocomiicoccus	0.0941	0.0000	0.0000	0.0000	40.0	0.0	-6.639	63	0.0105	0.0945	-0.400	Exploratory trend (0.05 ≤ q < 0.10)
[Ruminococcus] gauvreauii group	0.0286	0.0000	0.0000	0.0000	40.0	0.0	-4.953	63	0.0105	0.0945	-0.400	Exploratory trend (0.05 ≤ q < 0.10)
Proteus	0.0169	0.6828	0.0000	0.0178	6.7	50.0	5.258	150	0.0137	0.1116	0.429	Not significant
Arthrobacter	0.7234	0.0040	0.0196	0.0000	53.3	14.3	-7.195	57	0.0145	0.1126	-0.457	Not significant
Flavobacterium	0.0692	0.0044	0.0516	0.0000	66.7	35.7	-3.723	54.5	0.0206	0.1524	-0.481	Not significant
Pasteurella	0.0635	2.2414	0.0210	0.3874	60.0	78.6	5.121	157	0.0225	0.1529	0.495	Not significant
Aerococcus	0.2765	0.0019	0.0051	0.0000	53.3	14.3	-6.619	60	0.0220	0.1529	-0.429	Not significant
Agrococcus	0.0522	0.0008	0.0000	0.0000	40.0	7.1	-4.917	67.5	0.0315	0.2054	-0.357	Not significant
Chryseobacterium	0.0663	0.0068	0.0000	0.0000	46.7	7.1	-3.123	66	0.0329	0.2065	-0.371	Not significant
Bacteroides	1.4948	6.0570	0.4767	3.0997	100.0	100.0	2.018	154	0.0343	0.2070	0.467	Not significant
Pseudoalteromonas	0.1992	0.0666	0.0610	0.0041	86.7	50.0	-1.567	58	0.0394	0.2293	-0.448	Not significant
Hungatella	0.0523	0.0065	0.0000	0.0000	40.0	7.1	-2.833	70.5	0.0481	0.2615	-0.329	Not significant
Bacteroidales UCG-001	0.0404	0.0034	0.0000	0.0000	40.0	7.1	-3.261	70.5	0.0481	0.2615	-0.329	Not significant
Enterococcus	1.2627	0.2958	0.1657	0.0000	73.3	35.7	-2.090	63	0.0577	0.2740	-0.400	Not significant
Dietzia	0.1441	0.0264	0.0922	0.0000	66.7	42.9	-2.407	63	0.0577	0.2740	-0.400	Not significant
Paracoccus	0.0635	0.0043	0.0081	0.0000	60.0	28.6	-3.608	65	0.0588	0.2740	-0.381	Not significant
Prevotellaceae UCG-003	0.0998	0.0056	0.0000	0.0000	46.7	14.3	-3.946	69	0.0588	0.2740	-0.343	Not significant
Kingella	0.0223	0.0035	0.0000	0.0000	46.7	14.3	-2.386	69	0.0588	0.2740	-0.343	Not significant
Facklamia	0.2722	0.0150	0.0632	0.0122	66.7	57.1	-4.096	63	0.0626	0.2742	-0.400	Not significant
Sphingobacterium	0.1015	0.0538	0.0330	0.0000	66.7	35.7	-0.905	64.5	0.0639	0.2742	-0.386	Not significant

Genus	Non-pneumonic mean (%)	Pneumonic mean (%)	Non-pneumonic median (%)	Pneumonic median (%)	Non-pneumonic prevalence (%)	Pneumonic prevalence (%)	Log2 fold change	Mann–Whitney U	p value	BH-adjusted q value	Rank-biserial effect size	Interpretation
Turicibacter	0.0516	0.0116	0.0422	0.0000	60.0	35.7	-2.068	65	0.0634	0.2742	-0.381	Not significant
Johnsonella	0.0062	0.0302	0.0000	0.0023	20.0	50.0	2.125	141	0.0677	0.2830	0.343	Not significant
Pseudomonas	15.9957	6.2191	0.3282	0.0994	100.0	85.7	-1.363	64	0.0771	0.3065	-0.390	Not significant
Vibrio	0.4520	0.1822	0.1194	0.0194	93.3	71.4	-1.306	64	0.0764	0.3065	-0.390	Not significant
Caviibacter	0.3921	1.9331	0.0992	0.5601	86.7	92.9	2.299	145	0.0846	0.3282	0.381	Not significant
Clostridia vadinBB60 group	0.0435	0.0244	0.0332	0.0086	80.0	57.1	-0.810	67	0.0966	0.3663	-0.362	Not significant
Corynebacterium	1.5858	0.5429	1.5106	0.2744	100.0	85.7	-1.545	68	0.1111	0.3720	-0.352	Not significant
Romboutsia	0.1961	0.0495	0.0885	0.0191	80.0	71.4	-1.965	68	0.1087	0.3720	-0.352	Not significant
[Eubacterium] coprostanoligenes group	0.2734	0.0463	0.0193	0.0061	93.3	64.3	-2.536	68.5	0.1146	0.3720	-0.348	Not significant
Microbacterium	0.0858	0.0128	0.0225	0.0000	53.3	28.6	-2.658	72	0.1126	0.3720	-0.314	Not significant
WCHB1-41	0.1017	0.0078	0.0000	0.0000	46.7	21.4	-3.556	73	0.1050	0.3720	-0.305	Not significant
Bibersteinia	0.0058	2.1608	0.0000	0.0000	20.0	42.9	8.314	135	0.1164	0.3720	0.286	Not significant
Family XIII AD3011 group	0.0253	0.0025	0.0000	0.0000	40.0	14.3	-2.919	75	0.1022	0.3720	-0.286	Not significant
Parasutterella	0.0263	0.0092	0.0000	0.0000	33.3	7.1	-1.420	79	0.1160	0.3720	-0.248	Not significant
Aggregatibacter	0.0868	0.0444	0.0141	0.0000	60.0	28.6	-0.954	72	0.1201	0.3763	-0.314	Not significant
Barnesiella	0.1116	0.0431	0.0141	0.0000	73.3	42.9	-1.351	71	0.1294	0.3906	-0.324	Not significant
Sphaerochaeta	0.3418	0.0300	0.0000	0.0000	40.0	14.3	-3.471	77	0.1277	0.3906	-0.267	Not significant
Olsenella	0.0595	0.0162	0.0128	0.0000	60.0	35.7	-1.815	73	0.1387	0.4110	-0.305	Not significant
Ruminococcus	0.3579	0.0500	0.0344	0.0042	80.0	50.0	-2.817	71.5	0.1414	0.4117	-0.319	Not significant
Parvimonas	0.0730	0.3683	0.0283	0.0928	60.0	71.4	2.319	138	0.1476	0.4220	0.314	Not significant
uncultured	0.4125	0.1707	0.1707	0.0709	86.7	92.9	-1.268	73	0.1690	0.4749	-0.305	Not significant
Saccharimonadaceae	0.2138	0.1202	0.1600	0.0458	100.0	78.6	-0.825	74	0.1829	0.5054	-0.295	Not significant
Agathobacter	0.3237	0.0664	0.0172	0.0000	60.0	28.6	-2.269	77	0.1884	0.5118	-0.267	Not significant
NK4A214 group	0.3351	0.0983	0.0210	0.0000	60.0	42.9	-1.759	77	0.2028	0.5418	-0.267	Not significant
Bifidobacterium	0.4892	0.0703	0.0644	0.0095	66.7	57.1	-2.783	78	0.2344	0.5833	-0.257	Not significant
Rothia	0.0867	0.0210	0.0254	0.0067	60.0	57.1	-2.000	78	0.2303	0.5833	-0.257	Not significant
Blautia	0.3544	0.0836	0.0281	0.0057	66.7	50.0	-2.071	78.5	0.2392	0.5833	-0.252	Not significant
Atopostipes	0.0253	0.0057	0.0000	0.0000	40.0	28.6	-1.984	82	0.2469	0.5833	-0.219	Not significant
Lachnoclostridium	0.0235	0.0195	0.0000	0.0000	46.7	21.4	-0.262	82	0.2469	0.5833	-0.219	Not significant
Roseburia	0.0326	0.0045	0.0000	0.0000	40.0	21.4	-2.627	82.5	0.2416	0.5833	-0.214	Not significant
Moraxella	0.0050	0.2256	0.0000	0.0000	20.0	35.7	5.238	127	0.2336	0.5833	0.210	Not significant
Saccharofermentans	0.2207	0.0081	0.0000	0.0000	33.3	14.3	-4.613	84	0.2334	0.5833	-0.200	Not significant
Vagococcus	1.1392	0.0151	0.0000	0.0000	33.3	14.3	-6.154	85	0.2570	0.5901	-0.190	Not significant
CPla-4 termite group	0.0109	0.0175	0.0000	0.0000	33.3	14.3	0.634	85	0.2570	0.5901	-0.190	Not significant
Oligella	0.0533	0.0143	0.0229	0.0105	53.3	50.0	-1.832	80.5	0.2663	0.6014	-0.233	Not significant
UCG-010	0.0928	0.0128	0.0038	0.0000	53.3	42.9	-2.767	81	0.2693	0.6014	-0.229	Not significant
Neisseria	0.4487	0.2454	0.1610	0.0486	86.7	78.6	-0.868	80	0.2838	0.6094	-0.238	Not significant
Abiotrophia	0.0871	0.0530	0.0419	0.0257	80.0	64.3	-0.707	80.5	0.2898	0.6094	-0.233	Not significant
Snodgrassella	0.1990	0.1476	0.0676	0.0057	73.3	50.0	-0.428	81	0.2916	0.6094	-0.229	Not significant
UCG-002	0.1551	0.0386	0.0344	0.0066	60.0	50.0	-1.981	81	0.2824	0.6094	-0.229	Not significant
Prevotellaceae UCG-001	0.1627	0.0169	0.0000	0.0000	40.0	21.4	-3.197	84.5	0.2871	0.6094	-0.195	Not significant
Saccharimonadales	0.0919	0.1785	0.0423	0.0907	93.3	78.6	0.951	128.5	0.3149	0.6259	0.224	Not significant
Akkermansia	0.2302	0.0999	0.0803	0.0632	93.3	78.6	-1.197	81.5	0.3149	0.6259	-0.224	Not significant
Dorea	0.1422	0.0312	0.0000	0.0000	40.0	21.4	-2.155	85.5	0.3119	0.6259	-0.186	Not significant
Phyllobacterium	0.0228	0.0027	0.0000	0.0000	26.7	14.3	-2.691	88	0.3092	0.6259	-0.162	Not significant
Faecalibacterium	1.0443	0.2886	0.1192	0.0707	86.7	71.4	-1.852	83	0.3460	0.6308	-0.210	Not significant
Capnocytophaga	0.3546	0.2236	0.1530	0.0648	80.0	85.7	-0.663	83	0.3469	0.6308	-0.210	Not significant
Subdoligranulum	0.2322	0.0574	0.0204	0.0025	66.7	50.0	-1.999	83.5	0.3418	0.6308	-0.205	Not significant
Gemella	0.1660	0.1203	0.0676	0.0460	93.3	78.6	-0.461	83.5	0.3588	0.6308	-0.205	Not significant
Porphyromonas	0.7115	1.5378	0.2792	0.8383	100.0	92.9	1.111	126	0.3710	0.6308	0.200	Not significant
Lactobacillus	0.9681	0.8082	0.3726	0.1665	86.7	85.7	-0.260	84	0.3704	0.6308	-0.200	Not significant
F0332	0.0557	0.0482	0.0254	0.0000	66.7	42.9	-0.205	84	0.3484	0.6308	-0.200	Not significant
Gastranaerophilales	0.0318	0.0179	0.0101	0.0000	53.3	42.9	-0.798	84	0.3352	0.6308	-0.200	Not significant
Helicobacter	0.0449	0.0182	0.0129	0.0000	53.3	35.7	-1.257	84.5	0.3387	0.6308	-0.195	Not significant
Commensalibacter	0.1974	0.1248	0.0421	0.0000	60.0	42.9	-0.657	85	0.3664	0.6308	-0.190	Not significant
Bergeyella	0.0143	0.1145	0.0000	0.0059	40.0	50.0	2.918	124.5	0.3634	0.6308	0.186	Not significant
Carnobacterium	0.1303	0.0058	0.0000	0.0000	26.7	14.3	-4.279	89	0.3394	0.6308	-0.152	Not significant

Genus	Non-pneumonic mean (%)	Pneumonic mean (%)	Non-pneumonic median (%)	Pneumonic median (%)	Non-pneumonic prevalence (%)	Pneumonic prevalence (%)	Log2 fold change	Mann–Whitney U	p value	BH-adjusted q value	Rank-biserial effect size	Interpretation
[Eubacterium] nodatum group	0.0099	0.0102	0.0000	0.0000	33.3	14.3	0.046	89	0.3676	0.6308	-0.152	Not significant
Acetitomaculum	0.0285	0.0681	0.0000	0.0000	13.3	28.6	1.229	120	0.3715	0.6308	0.143	Not significant
Lautropia	0.0589	0.0236	0.0381	0.0025	53.3	50.0	-1.284	85.5	0.3789	0.6366	-0.186	Not significant
Phascolarctobacterium	0.0573	0.0319	0.0077	0.0000	60.0	42.9	-0.826	86	0.3915	0.6446	-0.181	Not significant
Desulfovibrio	0.0805	0.0048	0.0000	0.0000	33.3	21.4	-3.821	89	0.3905	0.6446	-0.152	Not significant
UCG-005	0.1747	0.0373	0.0356	0.0208	66.7	57.1	-2.198	86	0.4064	0.6535	-0.181	Not significant
Campylobacter	0.2092	0.4048	0.0880	0.1299	93.3	100.0	0.949	123	0.4450	0.6535	0.171	Not significant
Escherichia-Shigella	16.7291	17.3696	1.6426	0.8343	100.0	92.9	0.054	87	0.4450	0.6535	-0.171	Not significant
Prevotella	2.8447	1.9636	1.7721	0.8234	100.0	85.7	-0.535	87	0.4450	0.6535	-0.171	Not significant
Leptotrichia	1.5014	1.2197	0.8077	0.5183	100.0	85.7	-0.300	87	0.4450	0.6535	-0.171	Not significant
Veillonella	1.2369	0.9059	0.5221	0.3995	100.0	92.9	-0.449	87	0.4450	0.6535	-0.171	Not significant
Cardiobacterium	0.0500	0.0383	0.0115	0.0078	66.7	57.1	-0.376	87	0.4323	0.6535	-0.171	Not significant
Stenotrophomonas	0.0289	0.0261	0.0121	0.0000	53.3	35.7	-0.143	87.5	0.4161	0.6535	-0.167	Not significant
Butyricimonas	0.0511	0.0180	0.0000	0.0000	40.0	28.6	-1.457	89	0.4251	0.6535	-0.152	Not significant
Chloroplast	0.0086	0.0863	0.0000	0.0000	40.0	21.4	3.196	89.5	0.4247	0.6535	-0.148	Not significant
Succiniclasticum	0.3463	0.0636	0.0000	0.0000	26.7	14.3	-2.427	91	0.4054	0.6535	-0.133	Not significant
Intestinimonas	0.0271	0.0099	0.0000	0.0000	26.7	14.3	-1.370	92	0.4411	0.6535	-0.124	Not significant
[Eubacterium] hallii group	0.1731	0.0357	0.0000	0.0000	46.7	35.7	-2.248	89	0.4492	0.6537	-0.152	Not significant
Haemophilus	0.0514	0.0320	0.0000	0.0000	40.0	28.6	-0.669	90	0.4555	0.6571	-0.143	Not significant
Collinsella	0.1593	0.0595	0.0302	0.0109	73.3	78.6	-1.406	88	0.4684	0.6697	-0.162	Not significant
Megasphaera	0.0384	0.0225	0.0000	0.0000	46.7	35.7	-0.746	90	0.4790	0.6789	-0.143	Not significant
Erysipelotrichaceae UCG-003	0.0536	0.0187	0.0000	0.0000	33.3	21.4	-1.476	92	0.4887	0.6866	-0.124	Not significant
Clostridia UCG-014	0.5663	0.4985	0.2706	0.1872	100.0	85.7	-0.184	89	0.4987	0.6948	-0.152	Not significant
Christensenellaceae R-7 group	0.2898	0.1020	0.0414	0.0143	73.3	64.3	-1.498	90	0.5206	0.7131	-0.143	Not significant
Enterobacter	0.0170	0.0181	0.0000	0.0000	26.7	14.3	0.089	94	0.5176	0.7131	-0.105	Not significant
Muribaculaceae	0.3620	0.1030	0.0038	0.0019	53.3	50.0	-1.804	91.5	0.5471	0.7375	-0.129	Not significant
Oribacterium	0.0240	0.0445	0.0153	0.0000	53.3	42.9	0.864	92	0.5568	0.7375	-0.124	Not significant
Eikenella	0.0137	0.0131	0.0000	0.0000	40.0	28.6	-0.060	93	0.5540	0.7375	-0.114	Not significant
Comamonas	0.0104	0.0193	0.0000	0.0000	33.3	21.4	0.835	94	0.5608	0.7375	-0.105	Not significant
Leucobacter	0.0166	0.0083	0.0000	0.0000	26.7	21.4	-0.930	94.5	0.5611	0.7375	-0.100	Not significant
Granulicatella	0.0633	0.0723	0.0306	0.0424	66.7	78.6	0.190	118.5	0.5664	0.7386	0.129	Not significant
Dialister	0.2956	0.1592	0.0708	0.0542	100.0	85.7	-0.889	92	0.5853	0.7572	-0.124	Not significant
Clostridium sensu stricto 1	0.2107	0.1146	0.0766	0.0341	73.3	71.4	-0.873	93	0.6120	0.7855	-0.114	Not significant
Alistipes	0.2221	0.0583	0.0280	0.0215	66.7	64.3	-1.913	93.5	0.6240	0.7947	-0.110	Not significant
Lachnospiraceae NK4A136 group	0.0603	0.0130	0.0000	0.0000	26.7	21.4	-2.134	96.5	0.6419	0.8049	-0.081	Not significant
Anaerostipes	0.0410	0.0135	0.0000	0.0000	26.7	21.4	-1.537	96.5	0.6419	0.8049	-0.081	Not significant
Bacillus	0.0244	0.0151	0.0000	0.0000	33.3	28.6	-0.660	97	0.6898	0.8583	-0.076	Not significant
Butyricicoccus	0.0577	0.0209	0.0000	0.0000	46.7	35.7	-1.424	97	0.7142	0.8820	-0.076	Not significant
Treponema	0.1908	0.0747	0.0214	0.0095	80.0	64.3	-1.343	96.5	0.7242	0.8876	-0.081	Not significant
[Eubacterium] ruminantium group	0.0214	0.0047	0.0000	0.0000	20.0	28.6	-1.984	111	0.7492	0.9113	0.057	Not significant
Selenomonas	0.5772	0.5799	0.2658	0.2474	100.0	85.7	0.007	98	0.7766	0.9114	-0.067	Not significant
Actinomyces	0.5248	0.3601	0.1510	0.2023	100.0	92.9	-0.542	98	0.7767	0.9114	-0.067	Not significant
Candidatus Saccharimonas	0.0987	0.1178	0.0204	0.0354	66.7	78.6	0.253	111.5	0.7913	0.9114	0.062	Not significant
Tannerella	0.4179	0.4395	0.2008	0.1608	100.0	92.9	0.073	99	0.8103	0.9114	-0.057	Not significant
Parabacteroides	0.0650	0.0235	0.0000	0.0000	46.7	42.9	-1.431	99	0.7925	0.9114	-0.057	Not significant
Allorhizobium-Neorhizobium-Pararhizobium-Rhizobium	0.0247	0.0082	0.0000	0.0000	33.3	28.6	-1.483	99	0.7697	0.9114	-0.057	Not significant
Centipeda	0.0323	0.0289	0.0000	0.0000	40.0	35.7	-0.154	99.5	0.8025	0.9114	-0.052	Not significant
F082	0.1824	0.0363	0.0000	0.0000	33.3	28.6	-2.299	100	0.8107	0.9114	-0.048	Not significant
p-1088-a5 gut group	0.1433	0.0331	0.0000	0.0000	33.3	28.6	-2.084	100	0.8107	0.9114	-0.048	Not significant
Fusicatenibacter	0.1201	0.0219	0.0000	0.0000	26.7	28.6	-2.403	100	0.8031	0.9114	-0.048	Not significant
Tyzzerella	0.0163	0.0135	0.0000	0.0000	33.3	28.6	-0.255	100	0.8107	0.9114	-0.048	Not significant

Genus	Non-pneumonic mean (%)	Pneumonic mean (%)	Non-pneumonic median (%)	Pneumonic median (%)	Non-pneumonic prevalence (%)	Pneumonic prevalence (%)	Log2 fold change	Mann–Whitney U	p value	BH-adjusted q value	Rank-biserial effect size	Interpretation
F0058	0.0140	0.0244	0.0000	0.0000	46.7	35.7	0.762	101	0.8643	0.9650	-0.038	Not significant
Micrococcus	0.0336	0.0091	0.0000	0.0000	20.0	21.4	-1.777	102	0.8775	0.9665	-0.029	Not significant
Pantoea	0.0047	0.0174	0.0000	0.0000	20.0	21.4	1.710	108	0.8775	0.9665	0.029	Not significant
Klebsiella	0.2951	0.0219	0.0000	0.0000	20.0	28.6	-3.693	108	0.8845	0.9676	0.029	Not significant
Incertae Sedis	0.0155	0.0072	0.0000	0.0000	33.3	35.7	-1.002	102	0.8976	0.9716	-0.029	Not significant
Alloprevotella	0.1133	0.1848	0.0483	0.0564	80.0	85.7	0.701	108	0.9129	0.9716	0.029	Not significant
Lachnoanaerobaculum	0.0846	0.0864	0.0270	0.0332	66.7	71.4	0.030	108	0.9118	0.9716	0.029	Not significant
Stomatobaculum	0.0347	0.0370	0.0057	0.0042	53.3	50.0	0.090	102.5	0.9262	0.9716	-0.024	Not significant
[Ruminococcus] torques group	0.0694	0.0166	0.0000	0.0000	26.7	35.7	-2.003	107.5	0.9152	0.9716	0.024	Not significant
Rikenellaceae RC9 gut group	0.4968	0.1873	0.0058	0.0040	53.3	57.1	-1.403	103	0.9453	0.9716	-0.019	Not significant
Pseudopropionibacterium	0.1830	0.2039	0.0998	0.0424	86.7	85.7	0.156	103	0.9477	0.9716	-0.019	Not significant
Coprococcus	0.2020	0.0294	0.0000	0.0000	33.3	35.7	-2.741	103	0.9385	0.9716	-0.019	Not significant
Streptococcus	1.2740	1.5378	0.6158	0.6373	100.0	85.7	0.271	107	0.9478	0.9716	0.019	Not significant
TM7x	0.2934	0.4311	0.1620	0.2000	100.0	85.7	0.553	107	0.9478	0.9716	0.019	Not significant
Methanobrevibacter	0.1364	0.0528	0.0000	0.0000	33.3	35.7	-1.352	106	0.9795	0.9916	0.010	Not significant
Prevotellaceae NK3B31 group	0.0202	0.0906	0.0000	0.0000	33.3	28.6	2.112	104	0.9788	0.9916	-0.010	Not significant
Monoglobus	0.0776	0.0154	0.0000	0.0016	40.0	50.0	-2.264	104.5	1.0000	1.0000	-0.005	Not significant
Absconditabacteriales (SR1)	0.0357	0.0377	0.0178	0.0131	60.0	71.4	0.074	105	1.0000	1.0000	0.000	Not significant

Table S3. Complete ANCOM differential abundance results.

Differential abundance was assessed using ANCOM in QIIME 2. The W statistic represents the number of pairwise log-ratio tests rejecting the null hypothesis. Features with “Reject null hypothesis = TRUE” were considered differentially abundant.

Taxonomy	Domain	Phylum	Class	Order	Family	Genus	W statistic	Reject null hypothesis	ANCOM interpretation
d__Bacteria;p__Proteobacteria;c__Gammaproteobacteria;o__Pasteurellales;f__Pasteurellaceae;g__Mannheimia	Bacteria	Proteobacteria	Gammaproteobacteria	Pasteurellales	Pasteurellaceae	Mannheimia	209	TRUE	Differentially abundant
d__Bacteria;p__Actinobacteriota;c__Actinobacteria;o__Micrococcales;f__Micrococcaceae;g__Glutamicibacter	Bacteria	Actinobacteriota	Actinobacteria	Micrococcales	Micrococcaceae	Glutamicibacter	180	FALSE	Not differentially abundant
d__Bacteria;p__Firmicutes;c__Bacilli;o__Mycoplasmatales;f__Mycoplasmataceae;g__Mycoplasma	Bacteria	Firmicutes	Bacilli	Mycoplasmatales	Mycoplasmataceae	Mycoplasma	35	FALSE	Not differentially abundant
d__Bacteria;p__Fusobacteriota;c__Fusobacteriia;o__Fusobacteriales;f__Fusobacteriaceae;g__Fusobacterium	Bacteria	Fusobacteriota	Fusobacteriia	Fusobacteriales	Fusobacteriaceae	Fusobacterium	23	FALSE	Not differentially abundant
d__Bacteria;p__Proteobacteria;c__Gammaproteobacteria;o__Pseudomonadales;f__Moraxellaceae;g__Psychrobacter	Bacteria	Proteobacteria	Gammaproteobacteria	Pseudomonadales	Moraxellaceae	Psychrobacter	22	FALSE	Not differentially abundant
d__Bacteria;p__Actinobacteriota;c__Actinobacteria;o__Actinomycetales;f__Actinomycetaceae;g__Trueperella	Bacteria	Actinobacteriota	Actinobacteria	Actinomycetales	Actinomycetaceae	Trueperella	20	FALSE	Not differentially abundant
d__Bacteria;p__Proteobacteria;c__Gammaproteobacteria;o__Alteromonadales;f__Pseudoalteromonadaceae;g__Pseudoalteromonas	Bacteria	Proteobacteria	Gammaproteobacteria	Alteromonadales	Pseudoalteromonadaceae	Pseudoalteromonas	15	FALSE	Not differentially abundant
d__Bacteria;p__Firmicutes;c__Clostridia;o__Peptostreptococcales-Tissierellales;f__Peptostreptococcaceae;g__Peptostreptococcus	Bacteria	Firmicutes	Clostridia	Peptostreptococcales-Tissierellales	Peptostreptococcaceae	Peptostreptococcus	14	FALSE	Not differentially abundant
d__Bacteria;p__Actinobacteriota;c__Actinobacteria;o__Micrococcales;f__Brevibacteriaceae;g__Brevibacterium	Bacteria	Actinobacteriota	Actinobacteria	Micrococcales	Brevibacteriaceae	Brevibacterium	13	FALSE	Not differentially abundant
d__Bacteria;p__Bacteroidota;c__Bacteroidia;o__Bacteroidales;f__Bacteroidaceae;g__Bacteroides	Bacteria	Bacteroidota	Bacteroidia	Bacteroidales	Bacteroidaceae	Bacteroides	11	FALSE	Not differentially abundant
d__Bacteria;p__Firmicutes;c__Bacilli;o__Staphylococcales;f__Staphylococcaceae;g__Staphylococcus	Bacteria	Firmicutes	Bacilli	Staphylococcales	Staphylococcaceae	Staphylococcus	11	FALSE	Not differentially abundant
d__Bacteria;p__Fusobacteriota;c__Fusobacteriia;o__Fusobacteriales;f__Leptotrichiaceae;g__Streptobacillus	Bacteria	Fusobacteriota	Fusobacteriia	Fusobacteriales	Leptotrichiaceae	Streptobacillus	11	FALSE	Not differentially abundant
d__Bacteria;p__Actinobacteriota;c__Actinobacteria;o__Micrococcales;f__Dermabacteraceae;g__Brachybacterium	Bacteria	Actinobacteriota	Actinobacteria	Micrococcales	Dermabacteraceae	Brachybacterium	10	FALSE	Not differentially abundant
d__Bacteria;p__Firmicutes;c__Clostridia;o__Peptostreptococcales-Tissierellales;f__Peptostreptococcales-Tissierellales;g__Helcococcus	Bacteria	Firmicutes	Clostridia	Peptostreptococcales-Tissierellales	Peptostreptococcales-Tissierellales	Helcococcus	10	FALSE	Not differentially abundant
d__Bacteria;p__Firmicutes;c__Bacilli;o__Staphylococcales;f__Staphylococcaceae	Bacteria	Firmicutes	Bacilli	Staphylococcales	Staphylococcaceae	Jeotgalicoccus	9	FALSE	Not differentially abundant

Taxonomy	Domain	Phylum	Class	Order	Family	Genus	W statistic	Reject null hypothesis	ANCOM interpretation
;g__Jeotgalicoccus									
d__Bacteria;p__Firmicutes;c__Bacilli;o__Lactobacillales;__;__	Bacteria	Firmicutes	Bacilli	Lactobacillales			8	FALSE	Not differentially abundant
d__Bacteria;p__Proteobacteria;c__Gammaproteobacteria;o__Pasteurellales;f__Pasteurellaceae;g__Pasteurella	Bacteria	Proteobacteria	Gammaproteobacteria	Pasteurellales	Pasteurellaceae	Pasteurella	8	FALSE	Not differentially abundant
d__Bacteria;p__Firmicutes;c__Bacilli;o__Bacillales;f__Planococcaceae;g__Planococcus	Bacteria	Firmicutes	Bacilli	Bacillales	Planococcaceae	Planococcus	8	FALSE	Not differentially abundant
d__Bacteria;p__Actinobacteriota;c__Actinobacteria;o__Propionibacteriales;f__Propionibacteriaceae;g__Tessaracoccus	Bacteria	Actinobacteriota	Actinobacteria	Propionibacteriales	Propionibacteriaceae	Tessaracoccus	8	FALSE	Not differentially abundant
d__Bacteria;p__Actinobacteriota;c__Actinobacteria;o__Micrococcales;f__Microbacteriaceae;g__Agrococcus	Bacteria	Actinobacteriota	Actinobacteria	Micrococcales	Microbacteriaceae	Agrococcus	7	FALSE	Not differentially abundant
d__Bacteria;p__Firmicutes;c__Clostridia;o__Peptostreptococcales-Tissierellales;f__Peptostreptococcales-Tissierellales;g__Peptoniphilus	Bacteria	Firmicutes	Clostridia	Peptostreptococcales-Tissierellales	Peptostreptococcales-Tissierellales	Peptoniphilus	7	FALSE	Not differentially abundant
d__Bacteria;p__Proteobacteria;c__Gammaproteobacteria;o__Vibrionales;f__Vibrionaceae;g__Vibrio	Bacteria	Proteobacteria	Gammaproteobacteria	Vibrionales	Vibrionaceae	Vibrio	7	FALSE	Not differentially abundant
d__Bacteria;p__Bacteroidota;c__Bacteroidia;o__Flavobacteriales;f__Flavobacteriaceae;g__Flavobacterium	Bacteria	Bacteroidota	Bacteroidia	Flavobacteriales	Flavobacteriaceae	Flavobacterium	6	FALSE	Not differentially abundant
d__Bacteria;p__Proteobacteria;c__Gammaproteobacteria;o__Enterobacterales;f__Morganellaceae;g__Proteus	Bacteria	Proteobacteria	Gammaproteobacteria	Enterobacterales	Morganellaceae	Proteus	6	FALSE	Not differentially abundant
d__Bacteria;p__Firmicutes;c__Bacilli;o__Lactobacillales;f__Aerococcaceae;g__Aerococcus	Bacteria	Firmicutes	Bacilli	Lactobacillales	Aerococcaceae	Aerococcus	5	FALSE	Not differentially abundant
d__Bacteria;p__Firmicutes;c__Bacilli;o__Lactobacillales;f__Carnobacteriaceae;g__Granulicatella	Bacteria	Firmicutes	Bacilli	Lactobacillales	Carnobacteriaceae	Granulicatella	5	FALSE	Not differentially abundant
d__Bacteria;p__Proteobacteria;c__Gammaproteobacteria;o__Pseudomonadales;f__Moraxellaceae;g__Acinetobacter	Bacteria	Proteobacteria	Gammaproteobacteria	Pseudomonadales	Moraxellaceae	Acinetobacter	4	FALSE	Not differentially abundant
d__Bacteria;p__Actinobacteriota;c__Actinobacteria;o__Micrococcales;f__Micrococcaceae;g__Arthrobacter	Bacteria	Actinobacteriota	Actinobacteria	Micrococcales	Micrococcaceae	Arthrobacter	4	FALSE	Not differentially abundant
d__Bacteria;p__Firmicutes;c__Clostridia;o__Lachnospirales;f__Lachnospiraceae;g__Hungatella	Bacteria	Firmicutes	Clostridia	Lachnospirales	Lachnospiraceae	Hungatella	4	FALSE	Not differentially abundant
d__Bacteria;p__Firmicutes;c__Clostridia;o__Lachnospirales;f__Lachnospiraceae;g__Johnsonella	Bacteria	Firmicutes	Clostridia	Lachnospirales	Lachnospiraceae	Johnsonella	4	FALSE	Not differentially abundant
d__Bacteria;p__Proteobacteria;c__Gammaproteobacteria;o__Burkholderiales;f__Neisseriaceae;g__Kingella	Bacteria	Proteobacteria	Gammaproteobacteria	Burkholderiales	Neisseriaceae	Kingella	4	FALSE	Not differentially abundant
d__Bacteria;p__Firmicutes;c__Bacilli;o__Staphylococcales;f__Staphylococcaceae;g__Nosocomiicoccus	Bacteria	Firmicutes	Bacilli	Staphylococcales	Staphylococcaceae	Nosocomiicoccus	4	FALSE	Not differentially abundant
d__Bacteria;p__Bacteroidota;c__Bacteroidia;o__Bacteroidales;f__Porphyromonad	Bacteria	Bacteroidota	Bacteroidia	Bacteroidales	Porphyromonadaceae	Porphyromonas	4	FALSE	Not differentially abundant

Taxonomy	Domain	Phylum	Class	Order	Family	Genus	W statistic	Reject null hypothesis	ANCOM interpretation
aceae;g__Porphyromonas									
d__Bacteria;p__Firmicutes;c__Bacilli;o__Erysipelotrichales;f__Erysipelotrichaceae;g__Turicibacter	Bacteria	Firmicutes	Bacilli	Erysipelotrichales	Erysipelotrichaceae	Turicibacter	4	FALSE	Not differentially abundant
d__Bacteria;p__Firmicutes;c__Clostridia;o__Lachnospirales;f__Lachnospiraceae;g__[Ruminococcus]_gauvreauii_group	Bacteria	Firmicutes	Clostridia	Lachnospirales	Lachnospiraceae	[Ruminococcus]_gauvreauii_group	4	FALSE	Not differentially abundant
d__Bacteria;p__Firmicutes;c__Clostridia;o__Peptostreptococcales-Tissierellales;f__Peptostreptococcaceae;__	Bacteria	Firmicutes	Clostridia	Peptostreptococcales-Tissierellales	Peptostreptococcaceae		3	FALSE	Not differentially abundant
d__Bacteria;p__Bacteroidota;c__Bacteroidia;o__Bacteroidales;f__Prevotellaceae;g__Alloprevotella	Bacteria	Bacteroidota	Bacteroidia	Bacteroidales	Prevotellaceae	Alloprevotella	3	FALSE	Not differentially abundant
d__Bacteria;p__Proteobacteria;c__Alphaproteobacteria;o__Rhizobiales;f__Rhizobiaceae;g__Aminobacter	Bacteria	Proteobacteria	Alphaproteobacteria	Rhizobiales	Rhizobiaceae	Aminobacter	3	FALSE	Not differentially abundant
d__Bacteria;p__Patescibacteria;c__Saccharimonadia;o__Saccharimonadales;f__Saccharimonadaceae;g__Candidatus_Saccharimonas	Bacteria	Patescibacteria	Saccharimonadia	Saccharimonadales	Saccharimonadaceae	Candidatus_Saccharimonas	3	FALSE	Not differentially abundant
d__Bacteria;p__Chloroflexi;c__Chloroflexia;o__Thermomicrobiales;f__JG30-KF-CM45;g__JG30-KF-CM45	Bacteria	Chloroflexi	Chloroflexia	Thermomicrobiales	JG30-KF-CM45	JG30-KF-CM45	3	FALSE	Not differentially abundant
d__Bacteria;p__Firmicutes;c__Bacilli;o__Lactobacillales;f__Carnobacteriaceae;g__Jeotgalibaca	Bacteria	Firmicutes	Bacilli	Lactobacillales	Carnobacteriaceae	Jeotgalibaca	3	FALSE	Not differentially abundant
d__Archaea;p__Halobacterota;c__Methanomicrobia;o__Methanomicrobiales;f__Methanocorpusculaceae;g__Methanocorpusculum	Archaea	Halobacterota	Methanomicrobia	Methanomicrobiales	Methanocorpusculaceae	Methanocorpusculum	3	FALSE	Not differentially abundant
d__Bacteria;p__Actinobacteriota;c__Actinobacteria;o__Micrococcales;f__Microbacteriaceae;g__Microbacterium	Bacteria	Actinobacteriota	Actinobacteria	Micrococcales	Microbacteriaceae	Microbacterium	3	FALSE	Not differentially abundant
d__Bacteria;p__Firmicutes;c__Clostridia;o__Peptostreptococcales-Tissierellales;f__Anaerovoracaceae;g__[Eubacterium]_nodatum_group	Bacteria	Firmicutes	Clostridia	Peptostreptococcales-Tissierellales	Anaerovoracaceae	[Eubacterium]_nodatum_group	3	FALSE	Not differentially abundant
d__Bacteria;p__Firmicutes;c__Negativicutes;o__Veillonellales-Selenomonadales;f__Selenomonadaceae;g__uncultured	Bacteria	Firmicutes	Negativicutes	Veillonellales-Selenomonadales	Selenomonadaceae	uncultured	3	FALSE	Not differentially abundant
d__Bacteria;p__Bacteroidota;c__Bacteroidia;o__Bacteroidales;f__Prevotellaceae;__	Bacteria	Bacteroidota	Bacteroidia	Bacteroidales	Prevotellaceae		2	FALSE	Not differentially abundant
d__Bacteria;p__Bacteroidota;c__Bacteroidia;o__Flavobacteriales;f__Flavobacteriaceae;__	Bacteria	Bacteroidota	Bacteroidia	Flavobacteriales	Flavobacteriaceae		2	FALSE	Not differentially abundant
d__Bacteria;p__Firmicutes;c__Bacilli;o__Lactobacillales;f__Aerococcaceae;__	Bacteria	Firmicutes	Bacilli	Lactobacillales	Aerococcaceae		2	FALSE	Not differentially abundant
d__Bacteria;p__Firmicutes;c__Negativicutes;o__Veillonellales-Selenomonadales;f__Selenomonadaceae;__	Bacteria	Firmicutes	Negativicutes	Veillonellales-Selenomonadales	Selenomonadaceae		2	FALSE	Not differentially abundant
d__Bacteria;p__Proteobacteria;__;__;__;__	Bacteria	Proteobacteria					2	FALSE	Not differentially abundant
d__Bacteria;p__Proteobact	Bacteria	Proteobacteria	Gammaproteobacteria	Burkholderiales	Comamonadaceae		2	FALSE	Not differentially abundant

Taxonomy	Domain	Phylum	Class	Order	Family	Genus	W statistic	Reject null hypothesis	ANCOM interpretation
eria;c__Gammaproteobacteria;o__Burkholderiales;f__Comamonadaceae;__									
d__Bacteria;p__Proteobacteria;c__Gammaproteobacteria;o__Burkholderiales;f__Neisseriaceae;__	Bacteria	Proteobacteria	Gammaproteobacteria	Burkholderiales	Neisseriaceae		2	FALSE	Not differentially abundant
d__Bacteria;p__Proteobacteria;c__Gammaproteobacteria;o__Pasteurellales;f__Pasteurellaceae;__	Bacteria	Proteobacteria	Gammaproteobacteria	Pasteurellales	Pasteurellaceae		2	FALSE	Not differentially abundant
d__Bacteria;p__Patescibacteria;c__Gracilibacteria;o__Absconditabacteriales_(SR1);f__Absconditabacteriales_(SR1);g__Absconditabacteriales_(SR1)	Bacteria	Patescibacteria	Gracilibacteria	Absconditabacteriales_(SR 1)	Absconditabacteriales_(SR 1)	Absconditabacteriales_(SR 1)	2	FALSE	Not differentially abundant
d__Bacteria;p__Firmicutes;c__Clostridia;o__Lachnospirales;f__Lachnospiraceae;g__Acetitomaculum	Bacteria	Firmicutes	Clostridia	Lachnospirales	Lachnospiraceae	Acetitomaculum	2	FALSE	Not differentially abundant
d__Bacteria;p__Actinobacteriota;c__Actinobacteria;o__Actinomycetales;f__Actinomycetaceae;g__Actinomyces	Bacteria	Actinobacteriota	Actinobacteria	Actinomycetales	Actinomycetaceae	Actinomyces	2	FALSE	Not differentially abundant
d__Bacteria;p__Bacteroidota;c__Bacteroidia;o__Bacteroidales;f__Rikenellaceae;g__Alistipes	Bacteria	Bacteroidota	Bacteroidia	Bacteroidales	Rikenellaceae	Alistipes	2	FALSE	Not differentially abundant
d__Bacteria;p__Proteobacteria;c__Alphaproteobacteria;o__Rhizobiales;f__Rhizobiaceae;g__Allorhizobium-Neorhizobium-Pararhizobium-Rhizobium	Bacteria	Proteobacteria	Alphaproteobacteria	Rhizobiales	Rhizobiaceae	Allorhizobium-Neorhizobium-Pararhizobium-Rhizobium	2	FALSE	Not differentially abundant
d__Bacteria;p__Firmicutes;c__Clostridia;o__Oscillospirales;f__Ruminococcaceae;g__Anaerofilum	Bacteria	Firmicutes	Clostridia	Oscillospirales	Ruminococcaceae	Anaerofilum	2	FALSE	Not differentially abundant
d__Bacteria;p__Firmicutes;c__Clostridia;o__Lachnospirales;f__Lachnospiraceae;g__Anaerostipes	Bacteria	Firmicutes	Clostridia	Lachnospirales	Lachnospiraceae	Anaerostipes	2	FALSE	Not differentially abundant
d__Bacteria;p__Actinobacteriota;c__Coriobacteriia;o__Coriobacteriales;f__Atopobiaceae;g__Atopobium	Bacteria	Actinobacteriota	Coriobacteriia	Coriobacteriales	Atopobiaceae	Atopobium	2	FALSE	Not differentially abundant
d__Bacteria;p__Firmicutes;c__Bacilli;o__Lactobacillales;f__Carnobacteriaceae;g__Atopostipes	Bacteria	Firmicutes	Bacilli	Lactobacillales	Carnobacteriaceae	Atopostipes	2	FALSE	Not differentially abundant
d__Bacteria;p__Firmicutes;c__Bacilli;o__Bacillales;f__Bacillaceae;g__Bacillus	Bacteria	Firmicutes	Bacilli	Bacillales	Bacillaceae	Bacillus	2	FALSE	Not differentially abundant
d__Bacteria;p__Bacteroidota;c__Bacteroidia;o__Flavobacteriales;f__Weeksellaceae;g__Bergeyella	Bacteria	Bacteroidota	Bacteroidia	Flavobacteriales	Weeksellaceae	Bergeyella	2	FALSE	Not differentially abundant
d__Bacteria;p__Proteobacteria;c__Gammaproteobacteria;o__Pasteurellales;f__Pasteurellaceae;g__Bibersteinia	Bacteria	Proteobacteria	Gammaproteobacteria	Pasteurellales	Pasteurellaceae	Bibersteinia	2	FALSE	Not differentially abundant
d__Bacteria;p__Proteobacteria;c__Alphaproteobacteria;o__Caulobacterales;f__Caulobacteraceae;g__Brevundimonas	Bacteria	Proteobacteria	Alphaproteobacteria	Caulobacterales	Caulobacteraceae	Brevundimonas	2	FALSE	Not differentially abundant
d__Bacteria;p__Firmicutes;c__Clostridia;o__Oscillospirales;f__Butyricicoccaceae;g__Butyricicoccus	Bacteria	Firmicutes	Clostridia	Oscillospirales	Butyricicoccaceae	Butyricicoccus	2	FALSE	Not differentially abundant
d__Bacteria;p__Bacteroidota;c__Bacteroidia;o__Bacteroidales;f__Marinifilaceae;g__Butyricimonas	Bacteria	Bacteroidota	Bacteroidia	Bacteroidales	Marinifilaceae	Butyricimonas	2	FALSE	Not differentially abundant
d__Bacteria;p__Planctomy	Bacteria	Planctomycetota	Planctomycetes	Pirellulales	Pirellulaceae	CPla-4_termite_group	2	FALSE	Not differentially abundant

Taxonomy	Domain	Phylum	Class	Order	Family	Genus	W statistic	Reject null hypothesis	ANCOM interpretation
cetota;c__Planctomycetes;o__Pirellulales;f__Pirellulaceae;g__CPla-4_termite_group									
d__Bacteria;p__Campilobacterota;c__Campylobacteria;o__Campylobacterales;f__Campylobacteraceae;g__Campylobacter	Bacteria	Campilobacterota	Campylobacteria	Campylobacterales	Campylobacteraceae	Campylobacter	2	FALSE	Not differentially abundant
d__Bacteria;p__Proteobacteria;c__Gammaproteobacteria;o__Cardiobacteriales;f__Cardiobacteriaceae;g__Cardiobacterium	Bacteria	Proteobacteria	Gammaproteobacteria	Cardiobacteriales	Cardiobacteriaceae	Cardiobacterium	2	FALSE	Not differentially abundant
d__Bacteria;p__Firmicutes;c__Bacilli;o__Erysipelotrichales;f__Erysipelatoclostridiaceae;g__Catenibacterium	Bacteria	Firmicutes	Bacilli	Erysipelotrichales	Erysipelatoclostridiaceae	Catenibacterium	2	FALSE	Not differentially abundant
d__Bacteria;p__Cyanobacteria;c__Cyanobacteriia;o__Chloroplast;f__Chloroplast;g__Chloroplast	Bacteria	Cyanobacteria	Cyanobacteriia	Chloroplast	Chloroplast	Chloroplast	2	FALSE	Not differentially abundant
d__Bacteria;p__Bacteroidota;c__Bacteroidia;o__Flavobacteriales;f__Weeksellaceae;g__Chryseobacterium	Bacteria	Bacteroidota	Bacteroidia	Flavobacteriales	Weeksellaceae	Chryseobacterium	2	FALSE	Not differentially abundant
d__Bacteria;p__Firmicutes;c__Clostridia;o__Clostridia_vadinBB60_group;f__Clostridia_vadinBB60_group;g__Clostridia_vadinBB60_group	Bacteria	Firmicutes	Clostridia	Clostridia_vadinBB60_group	Clostridia_vadinBB60_group	Clostridia_vadinBB60_group	2	FALSE	Not differentially abundant
d__Bacteria;p__Firmicutes;c__Clostridia;o__Clostridiales;f__Clostridiaceae;g__Clostridium_sensu_stricto_1	Bacteria	Firmicutes	Clostridia	Clostridiales	Clostridiaceae	Clostridium_sensu_stricto_1	2	FALSE	Not differentially abundant
d__Bacteria;p__Proteobacteria;c__Gammaproteobacteria;o__Burkholderiales;f__Comamonadaceae;g__Comamonas	Bacteria	Proteobacteria	Gammaproteobacteria	Burkholderiales	Comamonadaceae	Comamonas	2	FALSE	Not differentially abundant
d__Bacteria;p__Actinobacteriota;c__Actinobacteria;o__Propionibacteriales;f__Propionibacteriaceae;g__Cutibacterium	Bacteria	Actinobacteriota	Actinobacteria	Propionibacteriales	Propionibacteriaceae	Cutibacterium	2	FALSE	Not differentially abundant
d__Bacteria;p__Proteobacteria;c__Alphaproteobacteria;o__Rhizobiales;f__Devosiaceae;g__Devosia	Bacteria	Proteobacteria	Alphaproteobacteria	Rhizobiales	Devosiaceae	Devosia	2	FALSE	Not differentially abundant
d__Bacteria;p__Firmicutes;c__Negativicutes;o__Veillonellales-Selenomonadales;f__Veillonellaceae;g__Dialister	Bacteria	Firmicutes	Negativicutes	Veillonellales-Selenomonadales	Veillonellaceae	Dialister	2	FALSE	Not differentially abundant
d__Bacteria;p__Actinobacteriota;c__Actinobacteria;o__Corynebacteriales;f__Dietziaceae;g__Dietzia	Bacteria	Actinobacteriota	Actinobacteria	Corynebacteriales	Dietziaceae	Dietzia	2	FALSE	Not differentially abundant
d__Bacteria;p__Actinobacteriota;c__Coriobacteriia;o__Coriobacteriales;f__Eggerthellaceae;g__Eggerthella	Bacteria	Actinobacteriota	Coriobacteriia	Coriobacteriales	Eggerthellaceae	Eggerthella	2	FALSE	Not differentially abundant
d__Bacteria;p__Proteobacteria;c__Gammaproteobacteria;o__Burkholderiales;f__Neisseriaceae;g__Eikenella	Bacteria	Proteobacteria	Gammaproteobacteria	Burkholderiales	Neisseriaceae	Eikenella	2	FALSE	Not differentially abundant
d__Bacteria;p__Proteobacteria;c__Gammaproteobacteria;o__Enterobacterales;f__Enterobacteriaceae;g__Enterobacter	Bacteria	Proteobacteria	Gammaproteobacteria	Enterobacterales	Enterobacteriaceae	Enterobacter	2	FALSE	Not differentially abundant
d__Bacteria;p__Firmicutes;c__Bacilli;o__Erysipelotrichales;f__Erysipelotrichaceae;g__Erysipelothrix	Bacteria	Firmicutes	Bacilli	Erysipelotrichales	Erysipelotrichaceae	Erysipelothrix	2	FALSE	Not differentially abundant
d__Bacteria;p__Firmicutes;	Bacteria	Firmicutes	Bacilli	Erysipelotrichales	Erysipelatoclostridiaceae	Erysipelotrichaceae_UCG-	2	FALSE	Not differentially abundant

Taxonomy	Domain	Phylum	Class	Order	Family	Genus	W statistic	Reject null hypothesis	ANCOM interpretation
c__Bacilli;o__Erysipelotrichales;f__Erysipelatoclostridiaceae;g__Erysipelotrichaceae_UCG-003						003			
d__Bacteria;p__Bacteroidota;c__Bacteroidia;o__Bacteroidales;f__Paludibacteraceae;g__F0058	Bacteria	Bacteroidota	Bacteroidia	Bacteroidales	Paludibacteraceae	F0058	2	FALSE	Not differentially abundant
d__Bacteria;p__Firmicutes;c__Bacilli;o__Lactobacillales;f__Aerococcaceae;g__Facklamia	Bacteria	Firmicutes	Bacilli	Lactobacillales	Aerococcaceae	Facklamia	2	FALSE	Not differentially abundant
d__Bacteria;p__Firmicutes;c__Clostridia;o__Clostridia;f__Hungateiclostridiaceae;g__Fastidiosipila	Bacteria	Firmicutes	Clostridia	Clostridia	Hungateiclostridiaceae	Fastidiosipila	2	FALSE	Not differentially abundant
d__Bacteria;p__Actinobacteriota;c__Actinobacteria;o__Actinomycetales;f__Actinomycetaceae;g__Flaviflexus	Bacteria	Actinobacteriota	Actinobacteria	Actinomycetales	Actinomycetaceae	Flaviflexus	2	FALSE	Not differentially abundant
d__Bacteria;p__Firmicutes;c__Clostridia;o__Lachnospirales;f__Lachnospiraceae;g__Fusicatenibacter	Bacteria	Firmicutes	Clostridia	Lachnospirales	Lachnospiraceae	Fusicatenibacter	2	FALSE	Not differentially abundant
d__Bacteria;p__Cyanobacteria;c__Vampirivibrionia;o__Gastranaerophilales;f__Gastranaerophilales;g__Gastranaerophilales	Bacteria	Cyanobacteria	Vampirivibrionia	Gastranaerophilales	Gastranaerophilales	Gastranaerophilales	2	FALSE	Not differentially abundant
d__Bacteria;p__Firmicutes;c__Bacilli;o__Staphylococcales;f__Gemellaceae;g__Gemella	Bacteria	Firmicutes	Bacilli	Staphylococcales	Gemellaceae	Gemella	2	FALSE	Not differentially abundant
d__Bacteria;p__Campilobacterota;c__Campylobacteria;o__Campylobacterales;f__Helicobacteraceae;g__Helicobacter	Bacteria	Campilobacterota	Campylobacteria	Campylobacterales	Helicobacteraceae	Helicobacter	2	FALSE	Not differentially abundant
d__Bacteria;p__Firmicutes;c__Bacilli;o__Erysipelotrichales;f__Erysipelotrichaceae;g__Holdemanella	Bacteria	Firmicutes	Bacilli	Erysipelotrichales	Erysipelotrichaceae	Holdemanella	2	FALSE	Not differentially abundant
d__Bacteria;p__Firmicutes;c__Clostridia;o__Oscillospirales;f__Ruminococcaceae;g__Incertae_Sedis	Bacteria	Firmicutes	Clostridia	Oscillospirales	Ruminococcaceae	Incertae_Sedis	2	FALSE	Not differentially abundant
d__Bacteria;p__Firmicutes;c__Clostridia;o__Oscillospirales;f__Oscillospiraceae;g__Intestinimonas	Bacteria	Firmicutes	Clostridia	Oscillospirales	Oscillospiraceae	Intestinimonas	2	FALSE	Not differentially abundant
d__Bacteria;p__Proteobacteria;c__Gammaproteobacteria;o__Enterobacterales;f__Enterobacteriaceae;g__Klebsiella	Bacteria	Proteobacteria	Gammaproteobacteria	Enterobacterales	Enterobacteriaceae	Klebsiella	2	FALSE	Not differentially abundant
d__Bacteria;p__Firmicutes;c__Clostridia;o__Lachnospirales;f__Lachnospiraceae;g__Lachnoanaerobaculum	Bacteria	Firmicutes	Clostridia	Lachnospirales	Lachnospiraceae	Lachnoanaerobaculum	2	FALSE	Not differentially abundant
d__Bacteria;p__Firmicutes;c__Clostridia;o__Lachnospirales;f__Lachnospiraceae;g__Lachnospiraceae_NK4A136_group	Bacteria	Firmicutes	Clostridia	Lachnospirales	Lachnospiraceae	Lachnospiraceae_NK4A136_group	2	FALSE	Not differentially abundant
d__Bacteria;p__Actinobacteriota;c__Actinobacteria;o__Micrococcales;f__Microbacteriaceae;g__Leucobacter	Bacteria	Actinobacteriota	Actinobacteria	Micrococcales	Microbacteriaceae	Leucobacter	2	FALSE	Not differentially abundant
d__Bacteria;p__Proteobacteria;c__Gammaproteobacteria;o__Xanthomonadales;f__Xanthomonadaceae;g__Luteimonas	Bacteria	Proteobacteria	Gammaproteobacteria	Xanthomonadales	Xanthomonadaceae	Luteimonas	2	FALSE	Not differentially abundant
d__Bacteria;p__Desulfobacterota;c__Desulfovibrionia;o__Desulfovibrionales;f__Desulfovibrionaceae;g__Mailhella	Bacteria	Desulfobacterota	Desulfovibrionia	Desulfovibrionales	Desulfovibrionaceae	Mailhella	2	FALSE	Not differentially abundant

Taxonomy	Domain	Phylum	Class	Order	Family	Genus	W statistic	Reject null hypothesis	ANCOM interpretation
d__Archaea;p__Euryarchaeota;c__Methanobacteria;o__Methanobacteriales;f__Methanobacteriaceae;g__Methanosphaera	Archaea	Euryarchaeota	Methanobacteria	Methanobacteriales	Methanobacteriaceae	Methanosphaera	2	FALSE	Not differentially abundant
d__Bacteria;p__Proteobacteria;c__Alphaproteobacteria;o__Rhizobiales;f__Beijerinckiaceae;g__Methylobacterium-Methylorubrum	Bacteria	Proteobacteria	Alphaproteobacteria	Rhizobiales	Beijerinckiaceae	Methylobacterium-Methylorubrum	2	FALSE	Not differentially abundant
d__Bacteria;p__Proteobacteria;c__Alphaproteobacteria;o__Rickettsiales;f__Mitochondria;g__Mitochondria	Bacteria	Proteobacteria	Alphaproteobacteria	Rickettsiales	Mitochondria	Mitochondria	2	FALSE	Not differentially abundant
d__Bacteria;p__Firmicutes;c__Clostridia;o__Monoglobales;f__Monoglobaceae;g__Monoglobus	Bacteria	Firmicutes	Clostridia	Monoglobales	Monoglobaceae	Monoglobus	2	FALSE	Not differentially abundant
d__Bacteria;p__Proteobacteria;c__Gammaproteobacteria;o__Burkholderiales;f__Alcaligenaceae;g__Oligella	Bacteria	Proteobacteria	Gammaproteobacteria	Burkholderiales	Alcaligenaceae	Oligella	2	FALSE	Not differentially abundant
d__Bacteria;p__Firmicutes;c__Clostridia;o__Oscillospirales;f__Oscillospiraceae;g__Oscillospira	Bacteria	Firmicutes	Clostridia	Oscillospirales	Oscillospiraceae	Oscillospira	2	FALSE	Not differentially abundant
d__Bacteria;p__Proteobacteria;c__Gammaproteobacteria;o__Burkholderiales;f__Alcaligenaceae;g__Paenalcaligenes	Bacteria	Proteobacteria	Gammaproteobacteria	Burkholderiales	Alcaligenaceae	Paenalcaligenes	2	FALSE	Not differentially abundant
d__Bacteria;p__Proteobacteria;c__Gammaproteobacteria;o__Enterobacterales;f__Erwiniaceae;g__Pantoea	Bacteria	Proteobacteria	Gammaproteobacteria	Enterobacterales	Erwiniaceae	Pantoea	2	FALSE	Not differentially abundant
d__Bacteria;p__Bacteroidota;c__Bacteroidia;o__Bacteroidales;f__Tannerellaceae;g__Parabacteroides	Bacteria	Bacteroidota	Bacteroidia	Bacteroidales	Tannerellaceae	Parabacteroides	2	FALSE	Not differentially abundant
d__Bacteria;p__Proteobacteria;c__Alphaproteobacteria;o__Rhodobacterales;f__Rhodobacteraceae;g__Paracoccus	Bacteria	Proteobacteria	Alphaproteobacteria	Rhodobacterales	Rhodobacteraceae	Paracoccus	2	FALSE	Not differentially abundant
d__Bacteria;p__Proteobacteria;c__Gammaproteobacteria;o__Burkholderiales;f__Sutterellaceae;g__Parasutterella	Bacteria	Proteobacteria	Gammaproteobacteria	Burkholderiales	Sutterellaceae	Parasutterella	2	FALSE	Not differentially abundant
d__Bacteria;p__Bacteroidota;c__Bacteroidia;o__Sphingobacteriales;f__Sphingobacteriaceae;g__Pedobacter	Bacteria	Bacteroidota	Bacteroidia	Sphingobacteriales	Sphingobacteriaceae	Pedobacter	2	FALSE	Not differentially abundant
d__Bacteria;p__Firmicutes;c__Clostridia;o__Peptococcales;f__Peptococcaceae;g__Peptococcus	Bacteria	Firmicutes	Clostridia	Peptococcales	Peptococcaceae	Peptococcus	2	FALSE	Not differentially abundant
d__Bacteria;p__Firmicutes;c__Negativicutes;o__Acidaminococcales;f__Acidaminococcaceae;g__Phascolarctobacterium	Bacteria	Firmicutes	Negativicutes	Acidaminococcales	Acidaminococcaceae	Phascolarctobacterium	2	FALSE	Not differentially abundant
d__Bacteria;p__Proteobacteria;c__Alphaproteobacteria;o__Rhizobiales;f__Rhizobiaceae;g__Phyllobacterium	Bacteria	Proteobacteria	Alphaproteobacteria	Rhizobiales	Rhizobiaceae	Phyllobacterium	2	FALSE	Not differentially abundant
d__Bacteria;p__Proteobacteria;c__Gammaproteobacteria;o__Pseudomonadales;f__Pseudomonadaceae;g__Pseudomonas	Bacteria	Proteobacteria	Gammaproteobacteria	Pseudomonadales	Pseudomonadaceae	Pseudomonas	2	FALSE	Not differentially abundant
d__Bacteria;p__Actinobacteriota;c__Actinobacteria;o__Propionibacteriales;f__Propionibacteriaceae;g__Pseudopropionibacterium	Bacteria	Actinobacteriota	Actinobacteria	Propionibacteriales	Propionibacteriaceae	Pseudopropionibacterium	2	FALSE	Not differentially abundant
d__Bacteria;p__Synergistot	Bacteria	Synergistota	Synergistia	Synergistales	Synergistaceae	Pyramidobacter	2	FALSE	Not differentially abundant

Taxonomy	Domain	Phylum	Class	Order	Family	Genus	W statistic	Reject null hypothesis	ANCOM interpretation
a;c__Synergistia;o__Synergistales;f__Synergistaceae;g__Pyramidobacter									
d__Bacteria;p__Firmicutes;c__Clostridia;o__Peptostreptococcales-Tissierellales;f__Peptostreptococcaceae;g__Romboutsia	Bacteria	Firmicutes	Clostridia	Peptostreptococcales-Tissierellales	Peptostreptococcaceae	Romboutsia	2	FALSE	Not differentially abundant
d__Bacteria;p__Firmicutes;c__Clostridia;o__Lachnospirales;f__Lachnospiraceae;g__Roseburia	Bacteria	Firmicutes	Clostridia	Lachnospirales	Lachnospiraceae	Roseburia	2	FALSE	Not differentially abundant
d__Bacteria;p__Patescibacteria;c__Saccharimonadia;o__Saccharimonadales;f__Saccharimonadales;g__Saccharimonadales	Bacteria	Patescibacteria	Saccharimonadia	Saccharimonadales	Saccharimonadales	Saccharimonadales	2	FALSE	Not differentially abundant
d__Bacteria;p__Firmicutes;c__Bacilli;o__Staphylococcales;f__Staphylococcaceae;g__Salinicoccus	Bacteria	Firmicutes	Bacilli	Staphylococcales	Staphylococcaceae	Salinicoccus	2	FALSE	Not differentially abundant
d__Bacteria;p__Firmicutes;c__Negativicutes;o__Veillonellales-Selenomonadales;f__Selenomonadaceae;g__Selenomonas	Bacteria	Firmicutes	Negativicutes	Veillonellales-Selenomonadales	Selenomonadaceae	Selenomonas	2	FALSE	Not differentially abundant
d__Bacteria;p__Firmicutes;c__Bacilli;o__Erysipelotrichales;f__Erysipelotrichaceae;g__Solobacterium	Bacteria	Firmicutes	Bacilli	Erysipelotrichales	Erysipelotrichaceae	Solobacterium	2	FALSE	Not differentially abundant
d__Bacteria;p__Proteobacteria;c__Alphaproteobacteria;o__Sphingomonadales;f__Sphingomonadaceae;g__Sphingomonas	Bacteria	Proteobacteria	Alphaproteobacteria	Sphingomonadales	Sphingomonadaceae	Sphingomonas	2	FALSE	Not differentially abundant
d__Bacteria;p__Firmicutes;c__Clostridia;o__Lachnospirales;f__Lachnospiraceae;g__Stomatobaculum	Bacteria	Firmicutes	Clostridia	Lachnospirales	Lachnospiraceae	Stomatobaculum	2	FALSE	Not differentially abundant
d__Bacteria;p__Proteobacteria;c__Gammaproteobacteria;o__Aeromonadales;f__Succinivibrionaceae;g__Succinivibrio	Bacteria	Proteobacteria	Gammaproteobacteria	Aeromonadales	Succinivibrionaceae	Succinivibrio	2	FALSE	Not differentially abundant
d__Bacteria;p__Patescibacteria;c__Saccharimonadia;o__Saccharimonadales;f__Saccharimonadaceae;g__TM7a	Bacteria	Patescibacteria	Saccharimonadia	Saccharimonadales	Saccharimonadaceae	TM7a	2	FALSE	Not differentially abundant
d__Bacteria;p__Patescibacteria;c__Saccharimonadia;o__Saccharimonadales;f__Saccharimonadaceae;g__TM7x	Bacteria	Patescibacteria	Saccharimonadia	Saccharimonadales	Saccharimonadaceae	TM7x	2	FALSE	Not differentially abundant
d__Bacteria;p__Bacteroidota;c__Bacteroidia;o__Bacteroidales;f__Tannerellaceae;g__Tannerella	Bacteria	Bacteroidota	Bacteroidia	Bacteroidales	Tannerellaceae	Tannerella	2	FALSE	Not differentially abundant
d__Bacteria;p__Firmicutes;c__Clostridia;o__Lachnospirales;f__Lachnospiraceae;g__Tyzzerella	Bacteria	Firmicutes	Clostridia	Lachnospirales	Lachnospiraceae	Tyzzerella	2	FALSE	Not differentially abundant
d__Bacteria;p__Firmicutes;c__Clostridia;o__Oscillospirales;f__Oscillospiraceae;g__UCG-003	Bacteria	Firmicutes	Clostridia	Oscillospirales	Oscillospiraceae	UCG-003	2	FALSE	Not differentially abundant
d__Bacteria;p__Planctomycetota;c__Phycisphaerae;o__Tepidisphaerales;f__WD2101_soil_group;g__WD2101_soil_group	Bacteria	Planctomycetota	Phycisphaerae	Tepidisphaerales	WD2101_soil_group	WD2101_soil_group	2	FALSE	Not differentially abundant
d__Bacteria;p__Bacteroidota;c__Bacteroidia;o__Flavobacteriales;f__Weeksellaceae;g__Weeksella	Bacteria	Bacteroidota	Bacteroidia	Flavobacteriales	Weeksellaceae	Weeksella	2	FALSE	Not differentially abundant
d__Bacteria;p__Firmicutes;	Bacteria	Firmicutes	Clostridia	Lachnospirales	Lachnospiraceae	[Eubacterium]_ruminantium	2	FALSE	Not differentially abundant

Taxonomy	Domain	Phylum	Class	Order	Family	Genus	W statistic	Reject null hypothesis	ANCOM interpretation
c__Clostridia;o__Lachnospirales;f__Lachnospiraceae;g__[Eubacterium]_ruminantium_group						_group			
d__Bacteria;p__Firmicutes;c__Clostridia;o__Lachnospirales;f__Lachnospiraceae;g__[Ruminococcus]_gnavus_group	Bacteria	Firmicutes	Clostridia	Lachnospirales	Lachnospiraceae	[Ruminococcus]_gnavus_group	2	FALSE	Not differentially abundant
d__Bacteria;p__Firmicutes;c__Clostridia;o__Lachnospirales;f__Lachnospiraceae;g__[Ruminococcus]_torques_group	Bacteria	Firmicutes	Clostridia	Lachnospirales	Lachnospiraceae	[Ruminococcus]_torques_group	2	FALSE	Not differentially abundant
d__Bacteria;p__Actinobacteriota;c__Coriobacteriia;o__Coriobacteriales;f__Eggerthellaceae;g__uncultured	Bacteria	Actinobacteriota	Coriobacteriia	Coriobacteriales	Eggerthellaceae	uncultured	2	FALSE	Not differentially abundant
d__Bacteria;p__Bacteroidota;c__Bacteroidia;o__Bacteroidales;f__Barnesiellaceae;g__uncultured	Bacteria	Bacteroidota	Bacteroidia	Bacteroidales	Barnesiellaceae	uncultured	2	FALSE	Not differentially abundant
d__Bacteria;p__Bacteroidota;c__Bacteroidia;o__Sphingobacteriales;f__Sphingobacteriaceae;g__uncultured	Bacteria	Bacteroidota	Bacteroidia	Sphingobacteriales	Sphingobacteriaceae	uncultured	2	FALSE	Not differentially abundant
d__Bacteria;p__Firmicutes;c__Bacilli;o__Lactobacillales;f__Aerococcaceae;g__uncultured	Bacteria	Firmicutes	Bacilli	Lactobacillales	Aerococcaceae	uncultured	2	FALSE	Not differentially abundant
d__Bacteria;p__Firmicutes;c__Clostridia;o__Lachnospirales;f__Lachnospiraceae;g__uncultured	Bacteria	Firmicutes	Clostridia	Lachnospirales	Lachnospiraceae	uncultured	2	FALSE	Not differentially abundant
d__Bacteria;p__Firmicutes;c__Clostridia;o__Oscillospirales;f__Oscillospiraceae;g__uncultured	Bacteria	Firmicutes	Clostridia	Oscillospirales	Oscillospiraceae	uncultured	2	FALSE	Not differentially abundant
d__Bacteria;p__Firmicutes;c__Clostridia;o__Peptococcales;f__Peptococcaceae;g__uncultured	Bacteria	Firmicutes	Clostridia	Peptococcales	Peptococcaceae	uncultured	2	FALSE	Not differentially abundant
d__Bacteria;p__Proteobacteria;c__Alphaproteobacteria;o__Rhodospirillales;f__uncultured;g__uncultured	Bacteria	Proteobacteria	Alphaproteobacteria	Rhodospirillales	uncultured	uncultured	2	FALSE	Not differentially abundant
d__Bacteria;p__Firmicutes;c__Clostridia;o__Lachnospirales;f__Lachnospiraceae;__	Bacteria	Firmicutes	Clostridia	Lachnospirales	Lachnospiraceae		1	FALSE	Not differentially abundant
d__Bacteria;p__Firmicutes;c__Clostridia;o__Oscillospirales;f__Oscillospiraceae;__	Bacteria	Firmicutes	Clostridia	Oscillospirales	Oscillospiraceae		1	FALSE	Not differentially abundant
d__Bacteria;p__Firmicutes;c__Clostridia;o__Oscillospirales;f__Ruminococcaceae;__	Bacteria	Firmicutes	Clostridia	Oscillospirales	Ruminococcaceae		1	FALSE	Not differentially abundant
d__Bacteria;p__Firmicutes;c__Bacilli;o__Lactobacillales;f__Aerococcaceae;g__Abiotrophia	Bacteria	Firmicutes	Bacilli	Lactobacillales	Aerococcaceae	Abiotrophia	1	FALSE	Not differentially abundant
d__Bacteria;p__Firmicutes;c__Clostridia;o__Lachnospirales;f__Lachnospiraceae;g__Agathobacter	Bacteria	Firmicutes	Clostridia	Lachnospirales	Lachnospiraceae	Agathobacter	1	FALSE	Not differentially abundant
d__Bacteria;p__Proteobacteria;c__Gammaproteobacteria;o__Pasteurellales;f__Pasteurellaceae;g__Aggregatibacter	Bacteria	Proteobacteria	Gammaproteobacteria	Pasteurellales	Pasteurellaceae	Aggregatibacter	1	FALSE	Not differentially abundant
d__Bacteria;p__Verrucomicrobiota;c__Verrucomicrobiae;o__Verrucomicrobiales;f__Akkermansiaceae;g__Akkermansia	Bacteria	Verrucomicrobiota	Verrucomicrobiae	Verrucomicrobiales	Akkermansiaceae	Akkermansia	1	FALSE	Not differentially abundant
d__Bacteria;p__Firmicutes;c__Bacilli;o__Erysipelotrich	Bacteria	Firmicutes	Bacilli	Erysipelotrichales	Erysipelatoclostridiaceae	Asteroleplasma	1	FALSE	Not differentially abundant

Taxonomy	Domain	Phylum	Class	Order	Family	Genus	W statistic	Reject null hypothesis	ANCOM interpretation
ales;f__Erysipelatoclostridiaceae;g__Asteroleplasma									
d__Bacteria;p__Bacteroidota;c__Bacteroidia;o__Bacteroidales;f__Bacteroidales_UCG-001;g__Bacteroidales_UCG-001	Bacteria	Bacteroidota	Bacteroidia	Bacteroidales	Bacteroidales_UCG-001	Bacteroidales_UCG-001	1	FALSE	Not differentially abundant
d__Bacteria;p__Bacteroidota;c__Bacteroidia;o__Bacteroidales;f__Barnesiellaceae;g__Barnesiella	Bacteria	Bacteroidota	Bacteroidia	Bacteroidales	Barnesiellaceae	Barnesiella	1	FALSE	Not differentially abundant
d__Bacteria;p__Actinobacteriota;c__Actinobacteria;o__Bifidobacteriales;f__Bifidobacteriaceae;g__Bifidobacterium	Bacteria	Actinobacteriota	Actinobacteria	Bifidobacteriales	Bifidobacteriaceae	Bifidobacterium	1	FALSE	Not differentially abundant
d__Bacteria;p__Firmicutes;c__Clostridia;o__Lachnospirales;f__Lachnospiraceae;g__Blautia	Bacteria	Firmicutes	Clostridia	Lachnospirales	Lachnospiraceae	Blautia	1	FALSE	Not differentially abundant
d__Bacteria;p__Bacteroidota;c__Bacteroidia;o__Flavobacteriales;f__Flavobacteriaceae;g__Capnocytophaga	Bacteria	Bacteroidota	Bacteroidia	Flavobacteriales	Flavobacteriaceae	Capnocytophaga	1	FALSE	Not differentially abundant
d__Bacteria;p__Firmicutes;c__Bacilli;o__Lactobacillales;f__Carnobacteriaceae;g__Carnobacterium	Bacteria	Firmicutes	Bacilli	Lactobacillales	Carnobacteriaceae	Carnobacterium	1	FALSE	Not differentially abundant
d__Bacteria;p__Fusobacteriota;c__Fusobacteriia;o__Fusobacteriales;f__Leptotrichiaceae;g__Caviibacter	Bacteria	Fusobacteriota	Fusobacteriia	Fusobacteriales	Leptotrichiaceae	Caviibacter	1	FALSE	Not differentially abundant
d__Bacteria;p__Firmicutes;c__Negativicutes;o__Veillonellales-Selenomonadales;f__Selenomonadaceae;g__Centipeda	Bacteria	Firmicutes	Negativicutes	Veillonellales-Selenomonadales	Selenomonadaceae	Centipeda	1	FALSE	Not differentially abundant
d__Bacteria;p__Firmicutes;c__Clostridia;o__Christensenellales;f__Christensenellaceae;g__Christensenellaceae_R-7_group	Bacteria	Firmicutes	Clostridia	Christensenellales	Christensenellaceae	Christensenellaceae_R-7_group	1	FALSE	Not differentially abundant
d__Bacteria;p__Firmicutes;c__Clostridia;o__Clostridia_UCG-014;f__Clostridia_UCG-014;g__Clostridia_UCG-014	Bacteria	Firmicutes	Clostridia	Clostridia_UCG-014	Clostridia_UCG-014	Clostridia_UCG-014	1	FALSE	Not differentially abundant
d__Bacteria;p__Actinobacteriota;c__Coriobacteriia;o__Coriobacteriales;f__Coriobacteriaceae;g__Collinsella	Bacteria	Actinobacteriota	Coriobacteriia	Coriobacteriales	Coriobacteriaceae	Collinsella	1	FALSE	Not differentially abundant
d__Bacteria;p__Proteobacteria;c__Alphaproteobacteria;o__Acetobacterales;f__Acetobacteraceae;g__Commensalibacter	Bacteria	Proteobacteria	Alphaproteobacteria	Acetobacterales	Acetobacteraceae	Commensalibacter	1	FALSE	Not differentially abundant
d__Bacteria;p__Firmicutes;c__Clostridia;o__Lachnospirales;f__Lachnospiraceae;g__Coprococcus	Bacteria	Firmicutes	Clostridia	Lachnospirales	Lachnospiraceae	Coprococcus	1	FALSE	Not differentially abundant
d__Bacteria;p__Actinobacteriota;c__Actinobacteria;o__Corynebacteriales;f__Corynebacteriaceae;g__Corynebacterium	Bacteria	Actinobacteriota	Actinobacteria	Corynebacteriales	Corynebacteriaceae	Corynebacterium	1	FALSE	Not differentially abundant
d__Bacteria;p__Desulfobacterota;c__Desulfovibrionia;o__Desulfovibrionales;f__Desulfovibrionaceae;g__Desulfovibrio	Bacteria	Desulfobacterota	Desulfovibrionia	Desulfovibrionales	Desulfovibrionaceae	Desulfovibrio	1	FALSE	Not differentially abundant
d__Bacteria;p__Firmicutes;c__Clostridia;o__Lachnospirales;f__Lachnospiraceae;g__Dorea	Bacteria	Firmicutes	Clostridia	Lachnospirales	Lachnospiraceae	Dorea	1	FALSE	Not differentially abundant
d__Bacteria;p__Firmicutes;c__Bacilli;o__Lactobacillales;f__Enterococcaceae;g__	Bacteria	Firmicutes	Bacilli	Lactobacillales	Enterococcaceae	Enterococcus	1	FALSE	Not differentially abundant

Taxonomy	Domain	Phylum	Class	Order	Family	Genus	W statistic	Reject null hypothesis	ANCOM interpretation
Enterococcus									
d__Bacteria;p__Proteobacteria;c__Gammaproteobacteria;o__Enterobacterales;f__Enterobacteriaceae;g__Escherichia-Shigella	Bacteria	Proteobacteria	Gammaproteobacteria	Enterobacterales	Enterobacteriaceae	Escherichia-Shigella	1	FALSE	Not differentially abundant
d__Bacteria;p__Actinobacteriota;c__Actinobacteria;o__Actinomycetales;f__Actinomycetaceae;g__F0332	Bacteria	Actinobacteriota	Actinobacteria	Actinomycetales	Actinomycetaceae	F0332	1	FALSE	Not differentially abundant
d__Bacteria;p__Bacteroidota;c__Bacteroidia;o__Bacteroidales;f__F082;g__F082	Bacteria	Bacteroidota	Bacteroidia	Bacteroidales	F082	F082	1	FALSE	Not differentially abundant
d__Bacteria;p__Firmicutes;c__Clostridia;o__Oscillospirales;f__Ruminococcaceae;g__Faecalibacterium	Bacteria	Firmicutes	Clostridia	Oscillospirales	Ruminococcaceae	Faecalibacterium	1	FALSE	Not differentially abundant
d__Bacteria;p__Firmicutes;c__Clostridia;o__Peptostreptococcales-Tissierellales;f__Anaerovoracaceae;g__Family_XIII_AD3011_group	Bacteria	Firmicutes	Clostridia	Peptostreptococcales-Tissierellales	Anaerovoracaceae	Family_XIII_AD3011_group	1	FALSE	Not differentially abundant
d__Bacteria;p__Proteobacteria;c__Gammaproteobacteria;o__Pasteurellales;f__Pasteurellaceae;g__Haemophilus	Bacteria	Proteobacteria	Gammaproteobacteria	Pasteurellales	Pasteurellaceae	Haemophilus	1	FALSE	Not differentially abundant
d__Bacteria;p__Firmicutes;c__Clostridia;o__Lachnospirales;f__Lachnospiraceae;g__Lachnoclostridium	Bacteria	Firmicutes	Clostridia	Lachnospirales	Lachnospiraceae	Lachnoclostridium	1	FALSE	Not differentially abundant
d__Bacteria;p__Firmicutes;c__Clostridia;o__Lachnospirales;f__Lachnospiraceae;g__Lachnospiraceae_ND3007_group	Bacteria	Firmicutes	Clostridia	Lachnospirales	Lachnospiraceae	Lachnospiraceae_ND3007_group	1	FALSE	Not differentially abundant
d__Bacteria;p__Firmicutes;c__Bacilli;o__Lactobacillales;f__Lactobacillaceae;g__Lactobacillus	Bacteria	Firmicutes	Bacilli	Lactobacillales	Lactobacillaceae	Lactobacillus	1	FALSE	Not differentially abundant
d__Bacteria;p__Proteobacteria;c__Gammaproteobacteria;o__Burkholderiales;f__Burkholderiaceae;g__Lautropia	Bacteria	Proteobacteria	Gammaproteobacteria	Burkholderiales	Burkholderiaceae	Lautropia	1	FALSE	Not differentially abundant
d__Bacteria;p__Fusobacteriota;c__Fusobacteriia;o__Fusobacteriales;f__Leptotrichiaceae;g__Leptotrichia	Bacteria	Fusobacteriota	Fusobacteriia	Fusobacteriales	Leptotrichiaceae	Leptotrichia	1	FALSE	Not differentially abundant
d__Bacteria;p__Firmicutes;c__Negativicutes;o__Veillonellales-Selenomonadales;f__Veillonellaceae;g__Megasphaera	Bacteria	Firmicutes	Negativicutes	Veillonellales-Selenomonadales	Veillonellaceae	Megasphaera	1	FALSE	Not differentially abundant
d__Archaea;p__Euryarchaeota;c__Methanobacteria;o__Methanobacteriales;f__Methanobacteriaceae;g__Methanobrevibacter	Archaea	Euryarchaeota	Methanobacteria	Methanobacteriales	Methanobacteriaceae	Methanobrevibacter	1	FALSE	Not differentially abundant
d__Bacteria;p__Actinobacteriota;c__Actinobacteria;o__Micrococcales;f__Micrococcaceae;g__Micrococcus	Bacteria	Actinobacteriota	Actinobacteria	Micrococcales	Micrococcaceae	Micrococcus	1	FALSE	Not differentially abundant
d__Bacteria;p__Proteobacteria;c__Gammaproteobacteria;o__Pseudomonadales;f__Moraxellaceae;g__Moraxella	Bacteria	Proteobacteria	Gammaproteobacteria	Pseudomonadales	Moraxellaceae	Moraxella	1	FALSE	Not differentially abundant
d__Bacteria;p__Bacteroidota;c__Bacteroidia;o__Bacteroidales;f__Muribaculaceae;g__Muribaculaceae	Bacteria	Bacteroidota	Bacteroidia	Bacteroidales	Muribaculaceae	Muribaculaceae	1	FALSE	Not differentially abundant
d__Bacteria;p__Firmicutes;c__Clostridia;o__Oscillospirales;f__Oscillospiraceae;g__NK4A214_group	Bacteria	Firmicutes	Clostridia	Oscillospirales	Oscillospiraceae	NK4A214_group	1	FALSE	Not differentially abundant

Taxonomy	Domain	Phylum	Class	Order	Family	Genus	W statistic	Reject null hypothesis	ANCOM interpretation
d__Bacteria;p__Proteobacteria;c__Gammaproteobacteria;o__Burkholderiales;f__Neisseriaceae;g__Neisseria	Bacteria	Proteobacteria	Gammaproteobacteria	Burkholderiales	Neisseriaceae	Neisseria	1	FALSE	Not differentially abundant
d__Bacteria;p__Actinobacteriota;c__Coriobacteriia;o__Coriobacteriales;f__Atopobiaceae;g__Olsenella	Bacteria	Actinobacteriota	Coriobacteriia	Coriobacteriales	Atopobiaceae	Olsenella	1	FALSE	Not differentially abundant
d__Bacteria;p__Firmicutes;c__Clostridia;o__Lachnospirales;f__Lachnospiraceae;g__Oribacterium	Bacteria	Firmicutes	Clostridia	Lachnospirales	Lachnospiraceae	Oribacterium	1	FALSE	Not differentially abundant
d__Bacteria;p__Firmicutes;c__Clostridia;o__Peptostreptococcales-Tissierellales;f__Peptostreptococcales-Tissierellales;g__Parvimonas	Bacteria	Firmicutes	Clostridia	Peptostreptococcales-Tissierellales	Peptostreptococcales-Tissierellales	Parvimonas	1	FALSE	Not differentially abundant
d__Bacteria;p__Bacteroidota;c__Bacteroidia;o__Bacteroidales;f__Prevotellaceae;g__Prevotella	Bacteria	Bacteroidota	Bacteroidia	Bacteroidales	Prevotellaceae	Prevotella	1	FALSE	Not differentially abundant
d__Bacteria;p__Bacteroidota;c__Bacteroidia;o__Bacteroidales;f__Prevotellaceae;g__Prevotellaceae_NK3B31_group	Bacteria	Bacteroidota	Bacteroidia	Bacteroidales	Prevotellaceae	Prevotellaceae_NK3B31_group	1	FALSE	Not differentially abundant
d__Bacteria;p__Bacteroidota;c__Bacteroidia;o__Bacteroidales;f__Prevotellaceae;g__Prevotellaceae_UCG-001	Bacteria	Bacteroidota	Bacteroidia	Bacteroidales	Prevotellaceae	Prevotellaceae_UCG-001	1	FALSE	Not differentially abundant
d__Bacteria;p__Bacteroidota;c__Bacteroidia;o__Bacteroidales;f__Prevotellaceae;g__Prevotellaceae_UCG-003	Bacteria	Bacteroidota	Bacteroidia	Bacteroidales	Prevotellaceae	Prevotellaceae_UCG-003	1	FALSE	Not differentially abundant
d__Bacteria;p__Bacteroidota;c__Bacteroidia;o__Bacteroidales;f__Rikenellaceae;g__Rikenellaceae_RC9_gut_group	Bacteria	Bacteroidota	Bacteroidia	Bacteroidales	Rikenellaceae	Rikenellaceae_RC9_gut_group	1	FALSE	Not differentially abundant
d__Bacteria;p__Actinobacteriota;c__Actinobacteria;o__Micrococcales;f__Micrococcaceae;g__Rothia	Bacteria	Actinobacteriota	Actinobacteria	Micrococcales	Micrococcaceae	Rothia	1	FALSE	Not differentially abundant
d__Bacteria;p__Firmicutes;c__Clostridia;o__Oscillospirales;f__Ruminococcaceae;g__Ruminococcus	Bacteria	Firmicutes	Clostridia	Oscillospirales	Ruminococcaceae	Ruminococcus	1	FALSE	Not differentially abundant
d__Bacteria;p__Patescibacteria;c__Saccharimonadia;o__Saccharimonadales;f__Saccharimonadaceae;g__Saccharimonadaceae	Bacteria	Patescibacteria	Saccharimonadia	Saccharimonadales	Saccharimonadaceae	Saccharimonadaceae	1	FALSE	Not differentially abundant
d__Bacteria;p__Firmicutes;c__Clostridia;o__Clostridia;f__Hungateiclostridiaceae;g__Saccharofermentans	Bacteria	Firmicutes	Clostridia	Clostridia	Hungateiclostridiaceae	Saccharofermentans	1	FALSE	Not differentially abundant
d__Bacteria;p__Proteobacteria;c__Gammaproteobacteria;o__Burkholderiales;f__Neisseriaceae;g__Snodgrassella	Bacteria	Proteobacteria	Gammaproteobacteria	Burkholderiales	Neisseriaceae	Snodgrassella	1	FALSE	Not differentially abundant
d__Bacteria;p__Spirochaetota;c__Spirochaetia;o__Spirochaetales;f__Spirochaetaceae;g__Sphaerochaeta	Bacteria	Spirochaetota	Spirochaetia	Spirochaetales	Spirochaetaceae	Sphaerochaeta	1	FALSE	Not differentially abundant
d__Bacteria;p__Bacteroidota;c__Bacteroidia;o__Sphingobacteriales;f__Sphingobacteriaceae;g__Sphingobacterium	Bacteria	Bacteroidota	Bacteroidia	Sphingobacteriales	Sphingobacteriaceae	Sphingobacterium	1	FALSE	Not differentially abundant
d__Bacteria;p__Proteobacteria;c__Gammaproteobacteria;o__Xanthomonadales;f	Bacteria	Proteobacteria	Gammaproteobacteria	Xanthomonadales	Xanthomonadaceae	Stenotrophomonas	1	FALSE	Not differentially abundant

Taxonomy	Domain	Phylum	Class	Order	Family	Genus	W statistic	Reject null hypothesis	ANCOM interpretation
__Xanthomonadaceae;g__ Stenotrophomonas									
d__Bacteria;p__Firmicutes; c__Bacilli;o__Lactobacillales;f__Streptococcaceae;g__ Streptococcus	Bacteria	Firmicutes	Bacilli	Lactobacillales	Streptococcaceae	Streptococcus	1	FALSE	Not differentially abundant
d__Bacteria;p__Firmicutes; c__Clostridia;o__Oscillospirales;f__Ruminococcaceae ;g__Subdoligranulum	Bacteria	Firmicutes	Clostridia	Oscillospirales	Ruminococcaceae	Subdoligranulum	1	FALSE	Not differentially abundant
d__Bacteria;p__Firmicutes; c__Negativicutes;o__Acidaminococcales;f__Acidaminococcaceae;g__Succiniclasticum	Bacteria	Firmicutes	Negativicutes	Acidaminococcales	Acidaminococcaceae	Succiniclasticum	1	FALSE	Not differentially abundant
d__Bacteria;p__Spirochaetota;c__Spirochaetia;o__Spirochaetales;f__Spirochaetaceae;g__Treponema	Bacteria	Spirochaetota	Spirochaetia	Spirochaetales	Spirochaetaceae	Treponema	1	FALSE	Not differentially abundant
d__Bacteria;p__Firmicutes; c__Clostridia;o__Oscillospirales;f__Oscillospiraceae;g __UCG-002	Bacteria	Firmicutes	Clostridia	Oscillospirales	Oscillospiraceae	UCG-002	1	FALSE	Not differentially abundant
d__Bacteria;p__Firmicutes; c__Clostridia;o__Oscillospirales;f__Oscillospiraceae;g __UCG-005	Bacteria	Firmicutes	Clostridia	Oscillospirales	Oscillospiraceae	UCG-005	1	FALSE	Not differentially abundant
d__Bacteria;p__Firmicutes; c__Clostridia;o__Oscillospirales;f__UCG-010;g__UCG -010	Bacteria	Firmicutes	Clostridia	Oscillospirales	UCG-010	UCG-010	1	FALSE	Not differentially abundant
d__Bacteria;p__Firmicutes; c__Bacilli;o__Lactobacillales;f__Vagococcaceae;g__V agococcus	Bacteria	Firmicutes	Bacilli	Lactobacillales	Vagococcaceae	Vagococcus	1	FALSE	Not differentially abundant
d__Bacteria;p__Firmicutes; c__Negativicutes;o__Veillonellales-Selenomonadales;f__Veillonellaceae;g__Veillonella	Bacteria	Firmicutes	Negativicutes	Veillonellales-Selenomonadales	Veillonellaceae	Veillonella	1	FALSE	Not differentially abundant
d__Bacteria;p__Verrucomicrobiota;c__Kiritimatiellae;o __WCHB1-41;f__WCHB1-41;g__WCHB1-41	Bacteria	Verrucomicrobiota	Kiritimatiellae	WCHB1-41	WCHB1-41	WCHB1-41	1	FALSE	Not differentially abundant
d__Bacteria;p__Firmicutes; c__Clostridia;o__Oscillospirales;f__[Eubacterium]_coprostanoligenes_group;g__[Eubacterium]_coprostanoligenes_group	Bacteria	Firmicutes	Clostridia	Oscillospirales	[Eubacterium]_coprostanoligenes_group	[Eubacterium]_coprostanoligenes_group	1	FALSE	Not differentially abundant
d__Bacteria;p__Firmicutes; c__Clostridia;o__Lachnospirales;f__Lachnospiraceae;g __[Eubacterium]_hallii_group	Bacteria	Firmicutes	Clostridia	Lachnospirales	Lachnospiraceae	[Eubacterium]_hallii_group	1	FALSE	Not differentially abundant
d__Bacteria;p__Planctomycetota;c__Planctomycetes; o__Pirellulales;f__Pirellulaceae;g__p-1088-a5_gut_group	Bacteria	Planctomycetota	Planctomycetes	Pirellulales	Pirellulaceae	p-1088-a5_gut_group	1	FALSE	Not differentially abundant
d__Bacteria;p__Bacteroidota;c__Bacteroidia;o__Bacteroidales;f__uncultured;g__ uncultured	Bacteria	Bacteroidota	Bacteroidia	Bacteroidales	uncultured	uncultured	1	FALSE	Not differentially abundant
d__Bacteria;p__Firmicutes; c__Clostridia;o__Oscillospirales;f__Ruminococcaceae ;g__uncultured	Bacteria	Firmicutes	Clostridia	Oscillospirales	Ruminococcaceae	uncultured	1	FALSE	Not differentially abundant
